# Advanced gene nanocarriers/scaffolds in nonviral-mediated delivery system for tissue regeneration and repair

**DOI:** 10.1186/s12951-024-02580-8

**Published:** 2024-06-26

**Authors:** Wanheng Zhang, Yan Hou, Shiyi Yin, Qi Miao, Kyubae Lee, Xiaojian Zhou, Yongtao Wang

**Affiliations:** 1 Institute of Geriatrics, School of Medicine, Affiliated Nantong Hospital of Shanghai University (The Sixth People’s Hospital of Nantong), Shanghai University, Shanghai, 200444 China; 2 Joint International Research Laboratory of Biomaterials and Biotechnology in Organ Repair (Ministry of Education), Shanghai University, Shanghai, 200444 China; 3 Department of Pharmacy, China Pharmaceutical University, Nanjing, 210009 China; 4 Department of Biomedical Materials, Konyang University, Daejeon, 35365 Republic of Korea; 5 Department of Pediatrics, Shanghai General Hospital, School of Medicine, Shanghai Jiao Tong University, Shanghai, 200080 China

**Keywords:** Advanced gene/Drug nanocarriers, Nonviral-mediated delivery system, Gene therapy, Tissue regeneration, Cancer-resected tissue repair

## Abstract

Tissue regeneration technology has been rapidly developed and widely applied in tissue engineering and repair. Compared with traditional approaches like surgical treatment, the rising gene therapy is able to have a durable effect on tissue regeneration, such as impaired bone regeneration, articular cartilage repair and cancer-resected tissue repair. Gene therapy can also facilitate the production of in situ therapeutic factors, thus minimizing the diffusion or loss of gene complexes and enabling spatiotemporally controlled release of gene products for tissue regeneration. Among different gene delivery vectors and supportive gene-activated matrices, advanced gene/drug nanocarriers attract exceptional attraction due to their tunable physiochemical properties, as well as excellent adaptive performance in gene therapy for tissue regeneration, such as bone, cartilage, blood vessel, nerve and cancer-resected tissue repair. This paper reviews the recent advances on nonviral-mediated gene delivery systems with an emphasis on the important role of advanced nanocarriers in gene therapy and tissue regeneration.

## Introduction

Tissue defects including wound, infection and tumor removal have become a great challenge in clinic around the world [1–3]. Due to poor intrinsic repair capacity in somatic cells and differentiated cells, a myriad of tissues and organs are urgently desired for in vivo clinical transplantation to repair damaged tissues, organs and parts in recent years [4]. Therefore, tissue regeneration technology has been rapidly developed and widely applied in tissue engineering since it is firstly proposed by American National Science Foundation (NSF) in 1988 [5]. Tissue engineering is also defined to apply the basic criterion and manner of engineering science and life science to understand the structure-and-function relationship between physiological or pathological tissues and organs, and further develop artificial substitutes with biological activity to restore, maintain and improve the functions of tissues and organs [6]. Current strategies that are curative and work well consisting of autologous transplantation, allograft transplantation and artificial substitutes for tissue regeneration [7]. However, these methods suffer from some inevitable drawbacks, such as donor shortage and immune rejection response and so on [6, 8]. To circumambulate these problems, tremendous efforts have been contributed to explore novel tissue regeneration techniques with efficient repair ability and low immunogenicity [9, 10].

Gene therapy has provided a potential tactic to aid in the tissue regeneration and repair for destructive tissues [11, 12]. In this process, exogenous genes are delivered into targeting damage sites by gene carriers to achieve effective gene therapy of tissues and organs [13]. Generally, gene delivery carriers can be divided into viral and nonviral-mediated vectors [14]. Viral vectors can incorporate exogenous genes into DNA sequence of host cells to form stable transfection, consisting of genetic materials (RNA or DNA), protein-coated capsid and envelope of lipids surrounding the capsid [15]. Viral vectors with the advantages of stability, high transfection efficiency and extended expression window of related genes have been used in many clinical trials for cancer therapy, cardiovascular disease treatment and tissue regeneration [16–20]. Currently, the most commonly used viral vectors include retrovirus (RV), lentivirus (LV), adeno-associated virus (AAV), and adenovirus (AV) for gene delivery and therapy [21, 22]. Although viral-based gene delivery vector has gained a huge success in transfection efficiency and stability, the off-target effect, immune related adverse risk and restricted viral tropism are still inevitable events, which limit the further clinical application and development especially for AAV vectors [23–25]. These challenges motivate researchers to explore the alternative carriers to realize precise gene delivery. Advanced functional biomaterial-based gene delivery systems open a new insight for gene therapy by the means of encapsulation and immobilization of nonviral carriers [15]. Biomaterials-based gene delivery has attracted more and more attention in tissue regeneration and repair, due to their economic cost, low immunogenicity and more accurate local delivery ability [26]. Advanced biomaterials, including inorganic materials, polymer materials, lipids, proteins and peptides, exosomes and DNA nanostructures for gene therapy mainly have the characteristics of good biocompatibility, targeting accuracy ability and excellent degradability [27, 28]. Further, advanced biomaterials-based gene carriers have also been investigated and applied in tissue regeneration, such as bone, cartilage, blood vessel, nerve and cancer therapy [29–34]. Different tissue engineering application aspects with their specific features are matched with various nonviral biomaterials [35]. The biological outcome of gene delivery and therapy depends heavily on the selection of gene vectors. Better biomaterial-vector choice and collocation will pave a new way for gene therapy in the potential of tissue engineering and organ regeneration.

**Fig. 1:**
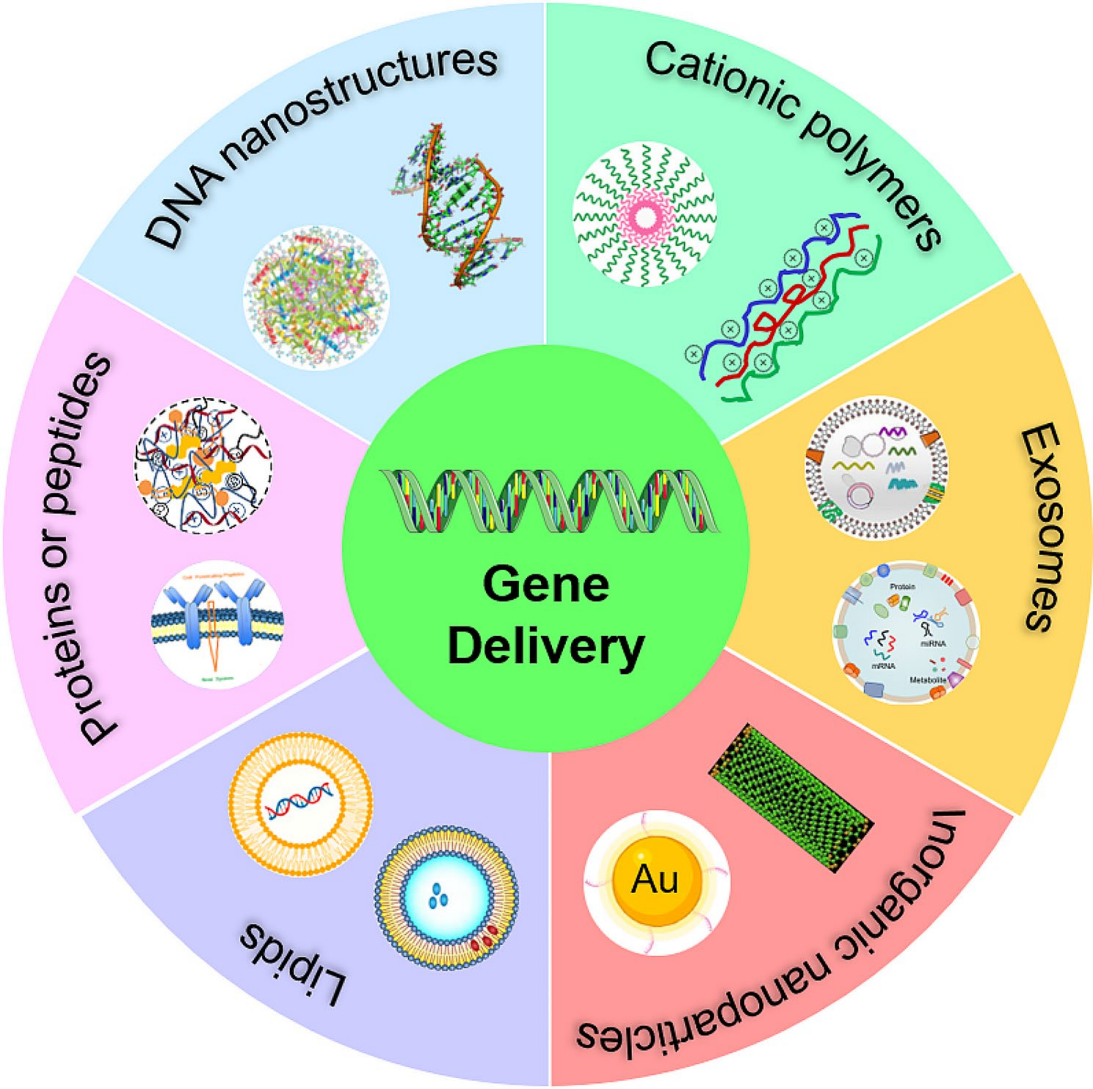
Nonviral-mediated advanced functional carriers for precise and targeting delivery of different nucleic acids, including dsDNA, ssDNA, siRNA, miRNA, mRNA and so on. Advanced biomaterials, such as lipid/lipid-like carriers, polymeric biomaterials, functional dendrimers, inorganic particles and exosomes, are intensively investigated for efficient gene delivery and therapy

In this review, we emphasize the nonviral-vector biomaterials developed for gene delivery and their application in tissue regeneration and repair (Fig. 1). Advanced nonviral biomaterials, including lipid/lipid-like carriers, polymeric biomaterials, functional dendrimers, inorganic particles and exosomes, carry the targeting genes, such as encoding DNA (double-stranded DNA (dsDNA), single-stranded DNA (ssDNA), plasmids DNA (pDNA)) and RNA (Messenger RNA (mRNA), MicroRNA (miRNA), small interfering RNA (siRNA)), into damaged tissues or sites for profitable gene therapy. On the other hand, we also highlight therapeutic applications of these advanced gene delivery materials in tissue regeneration, such as bone regeneration, cartilage regeneration, blood vessel regeneration, neuroregeneration and its broad potential in cancer-resected tissue repair and therapy. Finally, the strengths and limitations of different delivery vehicles are summarized in tissue regeneration and the review also shares our future perspectives in gene therapy and tissue regeneration. This will provide important information for advanced nonviral gene delivery materials in the potential of tissue engineering.

## Nonviral-mediated gene delivery systems

### The development of gene delivery and therapy

Gene delivery and therapy are considered to introduce exogenous normal or targeting genes (DNA or RNA) into objective cells to recompense for diseases aroused by defects and abnormal genes, so as to reach gene-related therapeutic purpose [36]. The conception of gene therapy is nominated in the early 1970s, the initial conceptualization is to interpose an accustomed gene to supersede a freakish gene [37, 38]. In 1990, the first clinical evaluation of a gene therapy approach to combating disease is launched at National Insititutes of Health (NIH) in the United States [39]. Since 2015, the worldwide gene therapy trade has flourished precipitately and also assessed that in 2025, the domestic and worldwide market of gene therapy will reach nearly 31 billion US dollars [40–42]. Therefore, gene therapy has been considered the third industrial revolution in bioloical medicine field after small molecule drugs and antibody drugs. Despite the rapid establishment of gene therapy, there are still rate-limiting steps, for example, the lack of efficient delivery system, weak sustained gene expression, and host immune reactions [43]. The Achilles’ heel of gene therapy is that how the targeting genes are effectively and accurately delivered into damaged sites for gene-mediated treatment. At present, gene delivery systems used in clinical trials are the basis for RV, LV, poxviruses, AV, AAV and herpes simplex virus (HSV) [44]. Nevertheless, inefficient delivery and transient gene expression are common issues with these vectors [45, 46]. The delivery system of advanced biomaterials will bring us a new dawn for desired gene therapy. Therefore, the development of drug delivery systems based on advanced functional biomaterials may serve as more effective drug and gene delivery vehicles for the treatment of harmful injury and malignant diseases. Moreover, we have also summarized the nonviral vectors under clinical trials or approved for human use currently (Table 1). The nonviral vectors have broadened human applications in the clinic.

**Table 1:** Nonviral vectors under clinical trials or approved for human use

Drug name	Modality	Disease or target	Vector property	Current clinical Phase	Vector component	Reference
Patisiran	siRNA	TTR	lipid Nps	Approved, 2018	DLin-MC3-DMA, DSPC, cholesterol, DMG-PEG 2000.	[47]
mRNA-1273	Vaccine	SARS-Cov-2	lipid Nps	Approved, 2021	DSPC, cholesterol, PEG2000-DMG, SM-102	[48]
BNT162b2	Vaccine	SARS-Cov-2	lipid Nps	Approved, 2021	ALC-0315, ALC-0159, DSPC, cholesterol	[49]
RO7198457	Vaccine	Colorectal Cancer	lipid Nps	Phase II	DOTMA and DOPE	[50]
CALAA-01	siRNA	RRM2	CD-containing polymer	Phase I, terminated	CD, PEG-AD, Tf-PEG-AD	[51]

*Notes* *TTR* Transthyretin, *PDAC* Pancreatic ductal adenocarcinoma, *RRM2* Ribonucleoside-diphosphate reductase subunit M2, *DSPC* 1,2-distearoyl-sn-glycero-3-phosphocholine, *DMG* dimyristoyl glycerol, *SM-102* 9-Heptadecanyl 8-{(2-hydroxyethyl) [6-oxo-6-(undecyloxy) hexyl]amino}octanoate, *ALC-0315* [(4-Hydroxybutyl) azanediyl]di (hexane-6,1-diyl) bis(2-hexyldecanoate), *ALC-0159* 2-[(polyethylene glycol)-2000]-N, N-ditetradecylacetamide, *DOTMA* 1,2-di-O-octadecenyl-3-trimethylammonium propane, *DOPE* 1,2-dioleoyl-sn-glycero-3-phosphoethanolamine, *CD* cyclodextrin, *AD* adamatane, *Tf* human transferrin

### Advanced gene nanocarriers/scaffolds in nonviral-mediated delivery systems

Many kinds of advanced biomaterials including lipid/lipid-like carriers, biopolymeric materials, functional dendrimers, inorganic biomaterials, exosomes and DNA nanostructures are designed and prepared to achieve efficient gene delivery and therapy [52, 53]. To improve gene delivery ability, there are various strategies based on targeting genes and advanced functional biomaterials to transfer exogenous genes and particles into impaired cells for transfection [54–56]. Common targeting genes contain encoding pDNA, mRNA, miRNA, siRNA, shRNA and circular RNAs (circRNAs) [57, 58]. Correspondingly, lipid carriers, cationic polymers, peptides, dendritic/branched materials and inorganic nanoparticles as gene delivery vectors are also performed well to enhance gene delivery and transfection. Moreover, extracellular vesicles (EVs) with the advantages of non-immunogenic, biodegradable, and biocompatible ability are considered as a promising delivery vehicle for various genetic therapeutics [59]. Lipid, lipid-like compounds and polymer compounds are one of the most intensively studied vectors for therapeutic gene delivery [60]. Herein, we will introduce these nonviral-mediated advanced biomaterials used for gene delivery and therapy in details.

#### Exosomes/DNA system

**Fig. 2:**
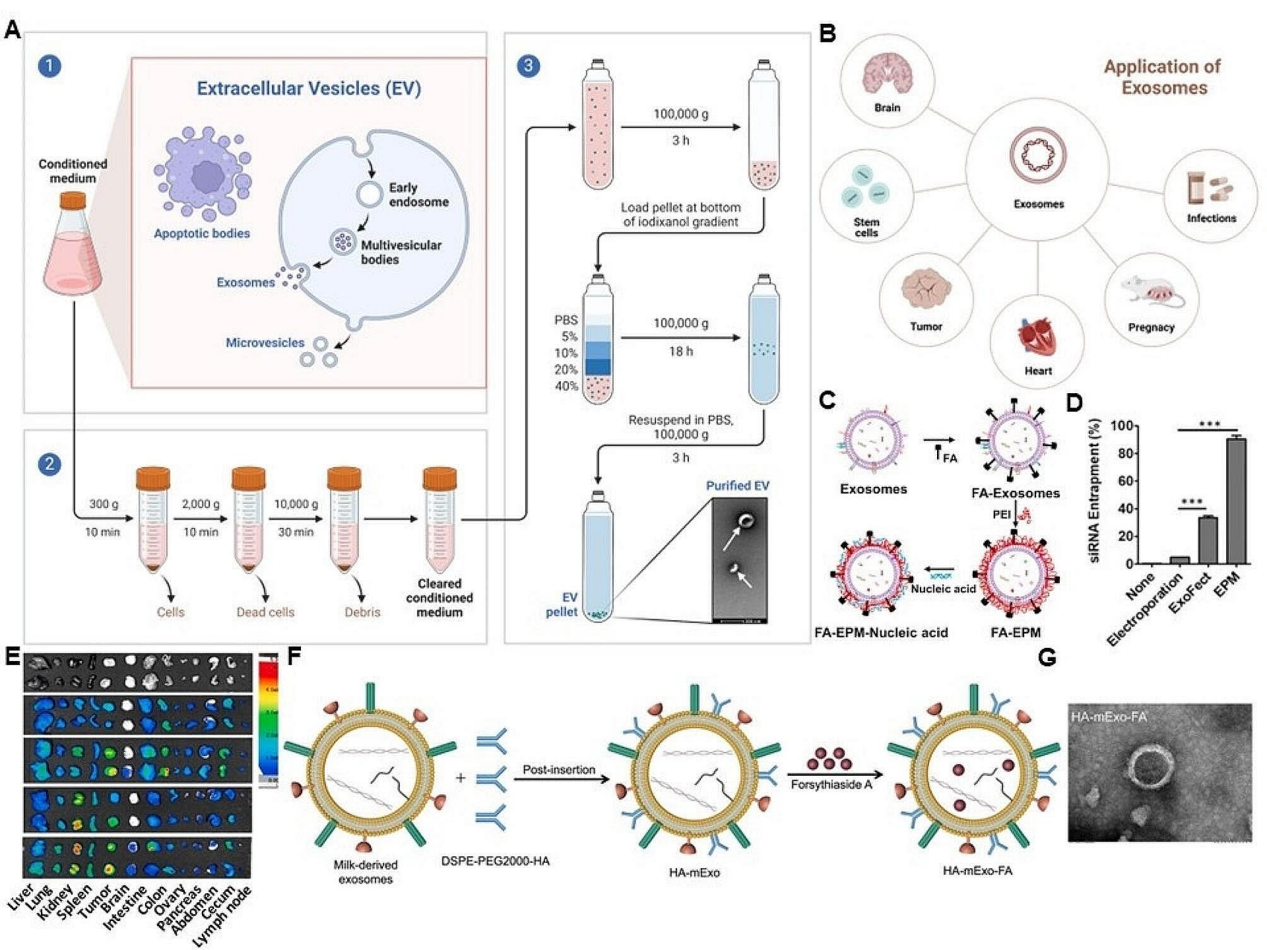
Exosomes as gene delivery vectors and their biomedical applications in gene therapy. (**A**) The production of biomimetic vesicles derived from cells through extrusion/filtration method. (**B**) The biomedical application of exosomes. (**C**) Representative illustration of FA covalently attached to the exosome-polyethyleneimine matrix (EPM) as a transfer carrier for nucleic acid delivery. (**D**) The entrapment ability of EPM with siKRAS and siRNA. (**E**) Tissue distribution of bovine colostrum exosomes and EPM, with and without FA-functionalized exosomes. (**C**-**E**) Reproduced with permission [70]. Copyright 2021, Elsevier. (**F**) Schematic graph of HA and FA loading into mExo. (**G**) Scanning electron microscope (SEM) photos of mExo and HA-mExo-FA. (**F**, **G**) Reproduced with permission [71]. Copyright 2023, Wiley

Extracellular vesicles (EVs) are regarded as small membrane vesicles to release into the extracellular matrix (ECM) and play an important role in regulating gene delivery and therapy [61, 62]. Exosomes with lipid-bilayer structure and the diameter of 40–160 nm are derived from different cells types, including stem cells, mast cells, epithelial cells, dendritic cells, B cells, T cells and cancer cells, to circulate in the extracellular environment [63, 64]. Exosomes from different cells have various functions and are applied in disease therapy [65, 66]. There are generally two forms of exosomes: One is biomimetic vesicles and the other is hybrid vescicles (Fig. 2A, B). For gene therapy, exosomes usually act as the efficient gene delivery system with versatile advantages, such as crossing biological barriers and genetically engineerable potential [67]. Yang and collaborators present an improved strategy to realize more efficient encapsulation and manageable release in recipient cells by encapsulating low-density lipoprotein receptor (Ldlr) mRNA into EVs [68]. There is an improved loading efficiency of Ldlr mRNA in EVs *via* MS2-MCP interaction [68]. Wood and collaborators also demonstrated that exosome-endogenous nanovesicles enable to transport short interfering (si)RNA to the brain in mice. GAPDH siRNA is specifically delivered by intravenously injected RVG-targeted exosomes to oligodendrocytes, microglia, neurons, causing a particular gene knockdown [69]. The integration of cell-derived EVs and synthesized polymers is further applied to complement targeting gene delivery capability. For instance, Gupta and collaborators successfully report a hybrid system of exosomes and PEI matrix (EPM) to provide high nucleic acid entrapment and protection from enzymatic degradation. The nanostructures can also enhance the specificity of delivery by combining a targeting ligand with no adverse response (Fig. 2C-E) [70]. In addition of the cell-derived EVs as gene delivery carriers, Li and collaborators develop a nanocarrier of CD44-specific ligand hyaluronic acid (HA)-modified milk-derived exosomes (mExo) encapsulated with folic acid (HA-mExo-FA). HA-mExo-FA could promote the apoptosis of activated LX2 cells and improve liver morphology and function alterations in zebrafish larvae (Fig. 2F, G) [71].

Exosomes are genetically engineered by the modification of polypeptide and nucleic acid to enhance the targeting gene therapy [63, 72]. For example, Liang and collaborators have fabricated an exosome-based chondrocyte-targeted miRNA (microRNA-140) delivery system for osteoarthritis (OA) therapy [73]. The chondrocyte-affinity peptide (CAP)-exosomes could carry miR-140 into profound cartilage sites through the dense mesochondrium to prevent cartilage-degrading proteases and slow down the OA progression. Moreover, exosomes can also transfer the CRISPR-Cas9 plasmids into targeting sites for gene therapy [74]. Kim and collaborators have reported that cancer-derived exosomes loaded CRISPR/Cas9 could restrain the expression of poly(ADP-ribose) polymerase-1 (PARP-1) to enhance the chemosensitivity of cisplatin and achieve satisfactory therapeutic effects in ovarian cancer tumors [75].

Based on structural and chemical traits of EVs and DNA, DNA nanostructure is constructed by the complementary nature of the four nucleotide bases as well [76]. By precisely controlling the DNA hybridization, stable branched DNA self-assembly, DNA programmability and desired DNA-sequence synthesis, DNA nanoparticles are fabricated by decorating DNA structures and sequences to deliver the designed genes for gene therapy [77, 78]. Moreover, EVs can also be modified by DNA aptamers or other molecules to realize the target activity or other functions for broad potentials in clinic [79, 80].

#### Lipids

Lipid-induced gene delivery platform works for gene therapy due to the effect of cationic lipid molecules [81, 82]. In principle, the positive charges on head group could interact with the negative charges of nucleic acids to establish targeting lipid/gene complexes [83, 84]. Then, the lipid/gene complexes could fuse with cell membrane and eventually enter into the targeting cell for gene silencing or integration in gene-related disease treatment [85]. The commercial Lipofectamine 2000 and Lipofectamine 3000 are the most commonly transfection reagent with their characteristics of simple operation, easy acquisition and high transfection efficiency [86, 87]. For instance, when these two reagents are applied to deliver mRNAs into dorsal root ganglion (DRG) neurons, the transfection efficiency can reach up to 25% under the assistance of Lipofectamine 2000 transfection reagent [88].

In addition, lipid and lipid-like carriers are also used for delivery of both small nucleic acids (siRNA, miRNA) and large ones (pDNA, mRNA) [89]. Kranz and collaborators have reported that RNA-lipoplexes (RNA-LPX) with adjusted net-like structures could enter into dendritic cell (DC) populations and macrophages to realize stable gene expression of the encoded antigen for cancer immunotherapy [90]. Rajala and collaborators have also designed and synthesized an artificial virus by using the functional nanoparticles of liposome-protamine-DNA (LPD) complexes and modifying with cell permeable peptide or nuclear localization signaling (NLS) peptide, to deliver the targeting engineered genes for eye disease treatment [91]. The results revealed that LPD could encourage effective gene delivery in a cell specific manner and achieve the long-term expression of Rpe65 gene to Rpe65-lacking mice, thus inducing blindness correction in vivo [91]. Due to the specific advantages of lipids in easier design for ideal properties by altering lipid components, particle size and surface charges, lipids have more broad application potentials in gene delivery and therapy [92].

#### Cationic polymers

**Fig. 3:**
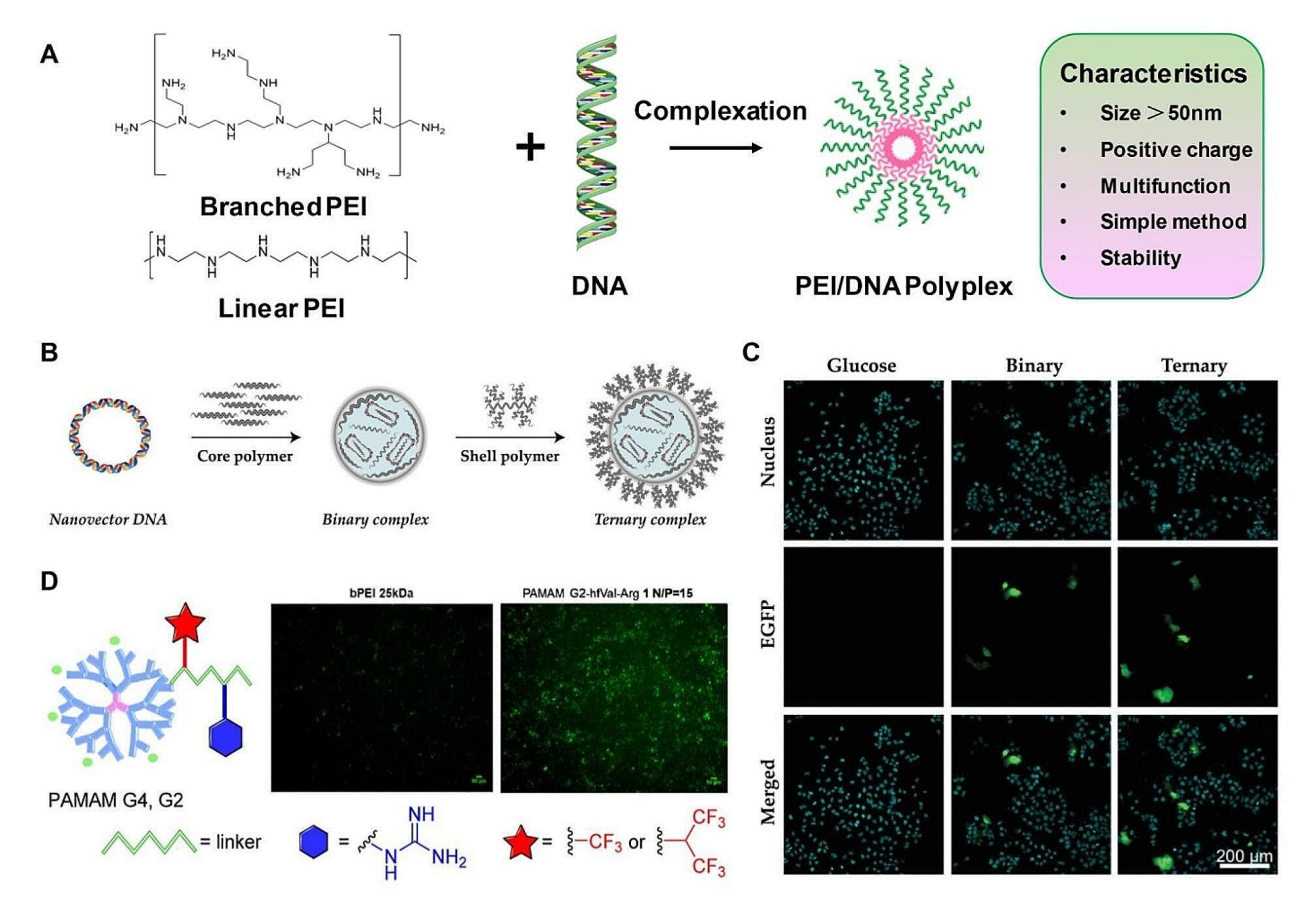
Cationic polymers as gene delivery vectors and their biomedical potentials. (**A**) PEI-based nanoparticle formation is designed by the admixing of PEI polymer derivatives (linear or branched) with nucleic acid cargos to encode for any genes of interest. (**B**) Schematic overview of ternary complex formation by sequential addition of core and shell polymer to the nanovector DNA. (**C**) In vitro evaluation of toxicity and transfection efficiency in HuH-7 cells. (**B**, **C**) Reproduced with permission [107]. Copyright 2023, Wiley. (**D**) Structural illustration of selectively fluorinated PAMAM–Arg conjugates and transfected cell micrographs. Reproduced with permission [108]. Copyright 2023, American Chemical Society

Similar to the mechanism of liposomes in gene delivery, cationic polymers also perform the delivery functions by the interaction with the negatively charged nucleic acids [93]. Cationic polymers with ionizable head groups can bind and condense DNA into small molecular structures to carry targeting genes for disease treatment [94]. Efficient transfer and cell internalization of DNAs are regulated through electrostatic interactions between cationic lipids and negatively charged nucleic acids or plasma membrane components [95]. For example, the amine-based cationic materials, including polylysine, polyamidoamine (PAMAM), PBAEs, poly(ethyleneimine) (PEI), cationic dendrimers, and chitosan, have also been explored as DNA vectors for gene delivery and therapy [96–98]. Among these cationic polymers, biofunctional PEI (50 kDa) supramolecular structures have demonstrated excellent transfection efficiency in vivo, compared to other amine-based polymers [99]. PEI serves as a classic and effective transfection agent for gene delivery. PEI possesses two structure forms, one is linear chain and another is branched chain, which can be synthesized at different condition (Fig. 3A) [100]. About 20% or 16.7% of the amino nitrogen is protonated at physiological pH [99, 101–103]. Besides, PEI has a high buffering capacity in a very wide pH range to escape from lysosomes and enhance the delivery productivity [104, 105]. Rohiwal and collaborators have constructed the targeting delivery strategy of CRISPR/Cas9 complexed PEI magnetic nanoparticles (MNPs) for gene therapy [106]. The 20-nm-in-diameter PEI-MNPs with sufficient colloidal stability show higher gene-editing efficiency, even compared with standard Lipofectamine transfection. To further improve the transfection efficiency, Lemaire and collaborators design and synthesize a depolymerized chitosan-polyethyleneimine DNA complex (dCS-PEI/DNA) for effective gene delivery (Fig. 3B). The results reveal that dCS-PEI/DNA complexes present the improved nucleic acid encapsulation ability, cellular uptake efficiency and transfer potency in human hepatoma HuH-7cells and murine primary hepatocytes (Fig. 3C) [107].

In addition of PEI-based gene carriers, PAMAM amino biomaterials are also applied as gene delivery vectors for efficient gene therapy. Volonterio and collaborators report the functionalization of the outer primary amines of PAMAM G2 and PAMAM G4 by building blocks bearing fluorinated moieties with a guanidino functional group. The obtained conjugates have the prominent advantages of improved transfection efficiency and negligible cytotoxicity (Fig. 3D) [108]. Nevertheless, poor biocompatibility, low biodegradability and high manufacturing costs greatly hinder their clinical applications for gene delivery and gene therapy.

#### Inorganic materials

Inorganic materials are also implemented by biofunctional modification as gene delivery carriers for purposeful gene therapy. The commonly used inorganic nanoparticles (NPs) consist of carbon-based NPs (quantum dots, graphene, carbon nanotubes and nanofibers), metal-based NPs (gold, copper, silver), semiconductor-based NPs (germanium, iron oxide) and silica NPs (SiO_2_) [109]. These inorganic NPs can be functionalized by biophysical or biochemical modification to adjust specific size of inorganic NPs and endow unique surface properties for precisely targeted gene delivery and therapy [110].

To enhance gene delivery ability of inorganic NPs, gold NPs are modified with nucleic acids *via* covalent or non-covalent conjugation through thiol moieties [111–114]. Dai and collaborators have fabricated the Janus gold/chitosan (J-Au-CS) NPs to realize fluorescence imaging-guided photothermal therapy (PTT) and PTT-enhanced gene therapy for cancer therapy [115]. Mesoporous silica nanoparticles (MSNs) with the diameter of 100–250 nm have also been used in gene delivery due to its good biocompatibility, porous size controllability and stable aqueous dispersion [116–118]. These MSNs are usually loaded with targeting genes by the means of weak non-covalent interactions [119]. Rasool and collaborators have also developed a thiolated, bioactive mesoporous silica NPs to improve the cell adhesion property in bone tissue regeneration [120]. The results reveal that MSN-SH groups can increase calcium deposition and induce osteogenesis by altering the bone-related gene expression [120]. Although inorganic materials play a vital role in gene delivery and therapy, especially in the cargo-loading ability and gene release rate, the toxicity and degradability are still unavoidable problems for gene delivery in vivo [121]. Some studies have demonstrated that gold and iron oxide nanoparticles are regarded as ideal non-toxic carriers for tissue regeneration and disease therapy [116, 122]. Moreover, the metabolism of these inorganic materials remains still a matter of concern in gene delivery.

#### Proteins and peptides

Protein and peptide-based formulations are also important delivery carriers, which usually consist of 5–30 amino acids with mostly hydrophobic and/or positively charged side chains [123]. Cell-penetrating peptides (CPPs) or homing peptides are typically representatives for gene delivery [124]. Positive charge in proteins and peptides can interact with negative charge by the means of covalent or non-covalent binding to enter into the cells [125]. Wang and collaborators have designed and prepared a library of glutathione (GSH)-responsive silica nanocapsules (SNCs), which could systemically deliver SNCs conjugated with glucose [126]. Under glycemic control, rabies virus glycoprotein peptide enables to bypass the intact blood-brain barrier (BBB) to achieve brain-wide transfer of various biologics and CRISPR genome editors can target various genes in both wild-type mice and Ai14 reporter mice [126]. Nevertheless, endowing CPPs with targeting ability and decreased toxicity remain great advantages for gene delivery of proteins and peptides in clinical potential.

In summary, DNA condensation, protection from nuclease, cell targeting, endosomal disruption, and nuclear translocation are the key factors for a successful gene delivery vector. For mechanism of biomaterials-based vectors entry into cells, most polymer/DNA complexes are uptaken into membrane-bound compartments by pinocytosis, adsorptive endocytosis, receptor-mediated endocytosis, or phagocytosis. After entering into the cells, they will escape from endosomal degradation using endosomolytic peptides or osmosis, and possibly obtain nuclear entry via an active nuclear skeleton mechanism. Therefore, cell-specific targeting ligands have been designed to link polymeric vectors to enhance the cell targeting efficiency by improving receptor-mediated endocytosis. For example, transferrin, monoclonal antibody, mannose, galactose, lactose, folic acid, low-density lipoproteins, and RGD peptides are the cell-specific targeting ligands to improve cellular internalization and gene transfection. In addition, biomaterial-mediated nonviral gene vectors also show respective advantages and disadvantages in gene delivery, including exosomes, lipids, cationic polymers, inorganic materials, proteins and peptides (Table 2).

**Table 2:** Advantages and disadvantages of biomaterial-mediated efficient nonviral gene delivery

Biomaterials-based vectors	Types	Advantages	Disadvantages
Exosomes	Extracellular vesicle	Wide source from various cell types Low immune inflammatory reaction Easy modification	Complicated operation Low production yield
Lipofectamine 2000/3000	Lipid	Good stability and low degradation Large-scale commercialization High gene transfection efficiency	Cytotoxicity in vivo Inflammatory reaction
PLL	Cationic polymer-based systems	Aggregation under physiological conditions	Low solubility of PLL/DNA complexes in aqueous media; cytotoxicity; undesirable effect of aggregation of PLL/DNA complex
PEI	Cationic polymer-based systems	Condense plasmids into colloidal particles that effectively transfect pDNA into a variety of cells both in vitro and in vivo; High transfection efficiency	Prone to aggregation and are toxic to the cell and cytotoxicity
Chitosan	Cationic polymer-based systems	Nontoxic biodegradable polysaccharide	Not so high efficiency
Dendrimers	Cationic polymer-based systems	The transfection efficiency dependens on the size, shape, and number of primary amine groups	Unstable
Quantum dots, graphene, carbon nanotubes or nanofibers	Carbon-based inorganic materials	High gene transfection efficiency Stability High production in industry	Aggregation Cytotoxicity Degradation
Gold, copper, silver	Metal-based inorganic materials	High transfection efficiency Simple modification	Cytotoxicity in vivo low degradation
Peptides with hydrophobic or positively charged side chains	Proteins and peptides	Low immune inflammatory reaction High gene transfection efficiency Nontoxic biodegradable materials	Complicated operation Unstable Biological safety

## Gene therapy for tissue regeneration

Gene therapy serves as a crucial role in modulating tissue regeneration and repair [127]. Regeneration is the process of self-renewal and repair by which a partial organism replaces lost or damaged tissue. Regenerative capacity widely varies across species, tissues, and life stages, and the process is also genetically programmed to accomplish injured tissue repair [128]. In the evolution of tissue regeneration, gene therapy can achieve point-to-point precise and targeted therapeutic effect of tissue repair through the transfer of genetic materials [129, 130]. The activity of DNA regulatory elements, transcription factors and chromatin regulators co-determine regeneration programming for tissue engineering [128]. For tissue regeneration, the targeting precision is critical to obtain the high efficiency and safety of gene therapy for disease diagnosis indications, such as tissue damage [130]. Yan and collaborators have found that tissue regeneration enhancer elements (TREEs) isolated from zebrafish can direct targeted, injury-associated gene expression from viral DNA vectors delivered systemically in small and large adult mammalian species [131]. These findings indicate that targeting gene delivery and therapy present huge potentials in tissue regeneration and repair.

### Tissue regeneration and repair

Tissue Engineering is a new discipline emerging in recent years, which belongs to the category of biological high technology [6, 132]. The term of tissue engineering is firstly proposed at the Bioengineering Group Meeting held by the American Science Foundation in Washington in 1987. It is officially defined in 1988 as: Combing the basic principles and new technologies of life science and engineering to correctly understand the normal and pathological conditions of mammals. On the basis of the relationship between tissue structure and function in the two states, it is an emerging discipline to study and develop biological substitutes for repairing, maintaining, and promoting the functions and forms of various tissues or organs of the human body after damage [6, 8]. Specially, tissue regeneration has ushered in vigorous process with the development of nanomedicine engineering and gene therapy in recent years.

Tissue engineering is divided into four aspects: Cell seeding and culture, advanced functional biomaterials for mimicking cell microniches, techniques for creating tissues and organs, and clinical utilization of tissue engineering [133, 134]. Currently, three approaches are constructed for tissue repair in clinical practice: (i) autologous tissue transplantation, (ii) allogeneic tissue transplantation, and (iii) the application of artificial substitutes [130, 135]. The three methods are respectively deficient and confronted with some mutual problems, such as immune rejection and donor shortage. The development of tissue engineering will fundamentally solve the problem of dysfunction or loss of treatment caused by tissue and organ defects [135]. The decisive essence of tissue engineering is to establish a three-dimensional (3D) space complex composed of cells and bioactive materials, which is radically different from traditional two-dimensional (2D) structures, such as cell culture patterns, nutritional supplement modes and 3D microenvironment niches [136, 137]. Therefore, the basic principles of tissue regeneration are: (1) simple reconstruction of tissue shape, structure and function to achieve permanent replacement, (2) abundant usage of least tissue cells to repair large tissue defects after in vitro culture and expansion, and (3) arbitrary geometry switching according to the tissue and organ defects to achieve a perfect shape repair [138].

### Advanced gene nanocarriers/scaffolds for gene therapy in tissue engineering

**Fig. 4:**
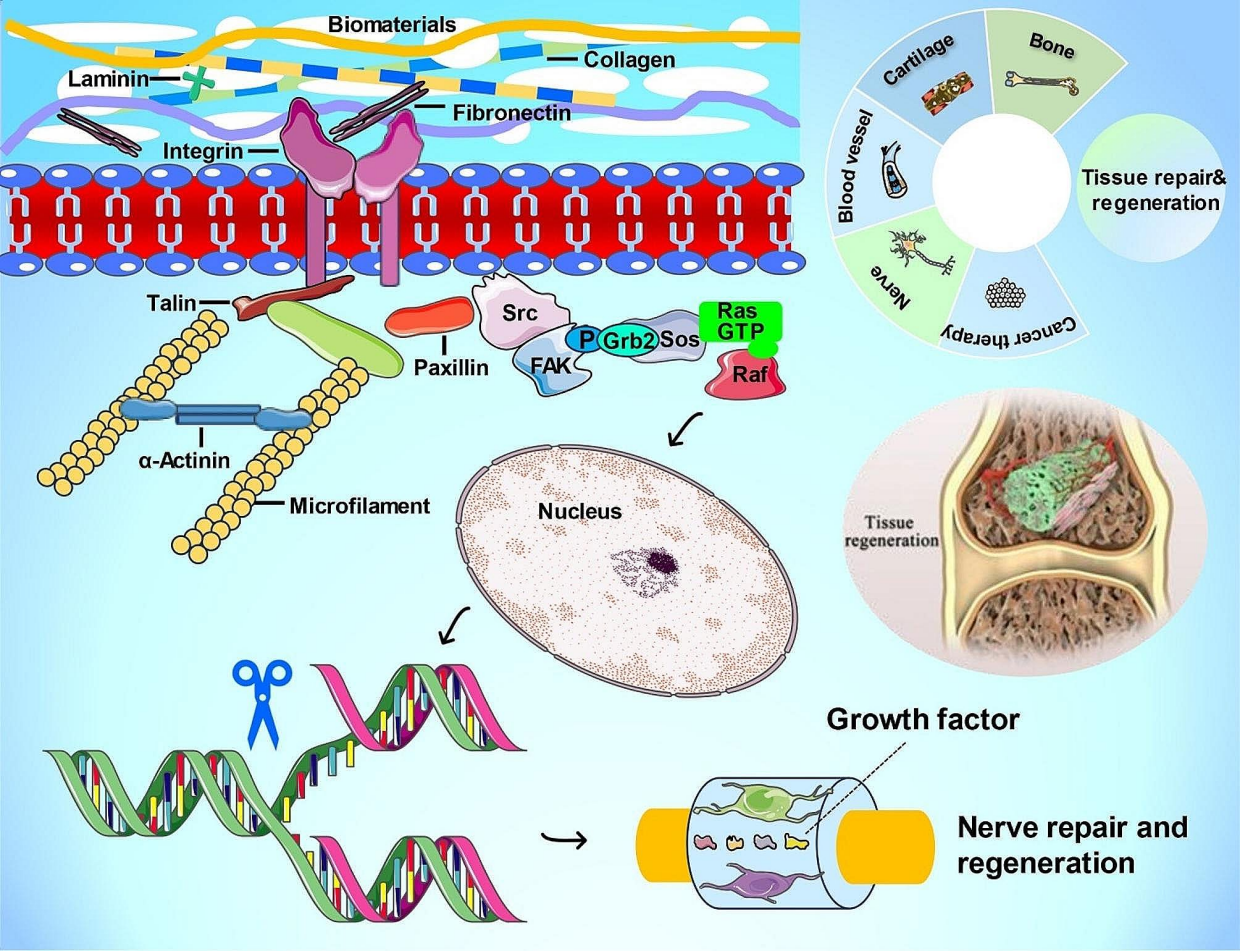
ECM remodeling and skeleton-mediated cellular nanomechanics enhance gene delivery through activation of force-sensing-related signaling pathways. Interaction of tissue regeneration related cells, including progenitor cells, iPSCs, ESCs and MSCs with advanced biomaterials to promote the expression of some functional proteins related to cell anchoring junction, such as collagen, fibronectin and laminin. These proteins can connect a series of intracellular anchor proteins, such as talin, vinculin and β-catenin for cell spreading and adhesion to alter the cytoskeleton and nuclear transcription to promote cell migration, division and differentiation and functional cell fate. Gene editing (knock in and knock out of targeting genes) also participate in this process to enhance gene therapy ability in tissue regeneration

Advanced functional biomaterials can act as gene carriers and regulators to deliver targeting genes or growth factors into corresponding damaged tissue sites to achieve tissue regeneration [139]. The tissue regeneration related cells, including progenitor cells, induced pluripotent stem cells (iPSCs), embryonic stem cells (ESCs) and mesenchymal stem cells (MSCs), can interact with the advanced biomaterials and promote the expression of some functional proteins related to cell anchoring junction (Fig. 4) [140–144]. The expression of collagen, fibronectin and laminin demonstrates the formation of adhesion anchorage between cells and extracellular matrix (ECM) [145, 146]. The transmembrane adhesion protein, integrin primarily binds collagen and fibronectin to produce adhesion sites for cell attachment, which is beneficial for migration, division and differentiation of stem cells in the process of tissue regeneration [147–150]. It can also connect a series of intracellular anchor proteins, such as talin, vinculin and β-catenin for cell spreading and adhesion [151–154]. Eventually, talin, vinculin, and β-catenin interacted with other molecules to form physical bridges, can alter the cytoskeleton and nuclear transcription to promote functional cell fate [155]. At the same time, gene editing (i.e. knock in and knock out of targeting genes) is also taken up in this process to enhance gene therapy in tissue regeneration [156, 157]. According to the discussion of gene therapy in tissue regeneration, gene therapy based on advanced functional gene delivery materials can play a vital role in tissue engineering and repair, such as bone regeneration, cartilage regeneration, blood vessel regeneration, nerve regeneration and repair (Table 3).

**Table 3:** Advanced gene/drug nanocarriers for gene therapy in tissue engineering

Application in tissue engineering	Therapeutic nucleic acids	Delivery vectors	Therapeutic outcomes	Ref.
Bone regeneration	pVEGF/pBMP2	Collagen-nanohydroxyapatite scaffold	Complete bridging of the defect after 4 weeks	[158]
Bone regeneration	miR-21a-5p	BMSCs derived exosomes	Promoted osteogenesis and osseointegration	[159]
Bone regeneration	Asp8-EM/miR-26a	Exosome-mimetics	Promotes bone regeneration in bone defect and osteoporosis models	[160]
Cartilage regeneration	pTGF-β1	Cupper sulfide@phosphatidylcholine	Efficient than commercial transfection agent	[161]
Cartilage regeneration	miR-224-5p	Transfected nanoparticles	Enhance miR-224-5p delivery ability	[162]
Cartilage regeneration	microRNA	Glycosaminoglycan-binding enhanced transduction (GET) NPs	Enhance cartilage defect repair	[163]
Blood vessel regeneration	Circ-Snhg11	Exosome-embedded GelMA (GelMA-Exo) hydrogels	Increase the survival of endothelial cells to maintain endothelial cell (EC) function	[164]
Blood vessel regeneration	miR-22	Laponite hydrogels	Reduce the infiltration of the macrophages and reverse the adverse vascular ECM remodeling	[165]
Blood vessel regeneration	miR-126	Poly(ethylene glycol)-b-poly(l-lactide-co-ε-caprolactone) (PELCL)	Improve endothelialization ability in vivo	[166]
Neuroregeneration	MiR-221/222	Cationic polymer (PDAPEI)	Promoted nerve regeneration after sciatic ne	[167]
Neuroregeneration	siRNA	polybutylcyanoacrylate nanoparticles (CaspNPs)	Restoration of central visual system damage	[168]
Neuroregeneration	Vector DNA	PEI/DNA polyplexes	Protein expression was detected in 82.8% ± 1.70% of DRG neurons	[169]

#### Advanced gene nanocarriers/scaffolds for gene therapy in bone regeneration

Long-distance bone defects, caused by trauma, tumor resection, and infection remain a worldwide concern due to the autologous regeneration insufficiency and alloimmune reaction. To date, the “gold standard” for bone defect repair is autologous bone grafting in clinic [170, 171]. Nonetheless, the extended treatment time at the graft donor site, significant pain, and increased treatment costs are the major limitations for bone regeneration [172]. On the other hand, bone allografts have the inevitable risk of immune rejection and bone size mismatch [173]. Moreover, metal bone implants have the drawbacks of poor osseointegration [174]. Therefore, the development of tissue-engineered bones based on the combination of efficient gene therapy has broad prospects in recent years.

Gene therapy is considered as an ideal way in many ways to enhance bone regeneration ability due to precisely targeting bone repair. Gene therapy is interpreted to transfer specific gene products to precise anatomical positions [175, 176]. In addition, transgene expression level and expression duration can be modulated with existing techniques [177]. For bone regeneration, genes of interest are transferred to the fracture locations, and expressed at suitable levels, and then inactivated after fracture healing [178]. The transmission of biological factors, primarily bone morphogenetic proteins (BMPs), has produced encouraging results in both animal and clinical studies [179]. A great deal of work has also been done on exploring previously defined growth factors and inventing new growth factors [158]. To enhance gene therapy capacity, gene delivery systems are designed from viral and nonviral vectors to tissue-engineered scaffolds to make significant progress [159]. Despite some public hesitation about gene therapy, this technology has huge potential applications to broaden our horizons to cure a wide range of human skeletal or musculoskeletal disorders.

Nonviral-mediated gene vectors contain chemical delivery and physical delivery methods [180]. Chemical delivery is mainly to complex cationic biomacromolecules or lipids with DNA/RNA to induce the electrochemical repulsion with cell membranes [160]. Physical methods of electroporation and sonoporation take advantage of electrical and mechanical energy respectively to build transient pores in cell membranes for efficient internalization of exogenous DNAs [56, 181]. Curtin and collaborators have reported a nonviral dual delivery system of vascular endothelial growth factors (VEGF) and bone morphogenetic protein 2 (BMP2) in a collagen-nanohydroxyapatite (nHA) scaffold, which reveals a marked 36-fold increase in bone formation in nHA scaffold versus the empty defect as early as 4 weeks post-implantation underlining the immense ability in bone regeneration [182]. In this scaffold, nHA particles and PEI are used to serve as nonviral vectors to achieve pDNA delivery for efficient gene therapy [182].

**Fig. 5:**
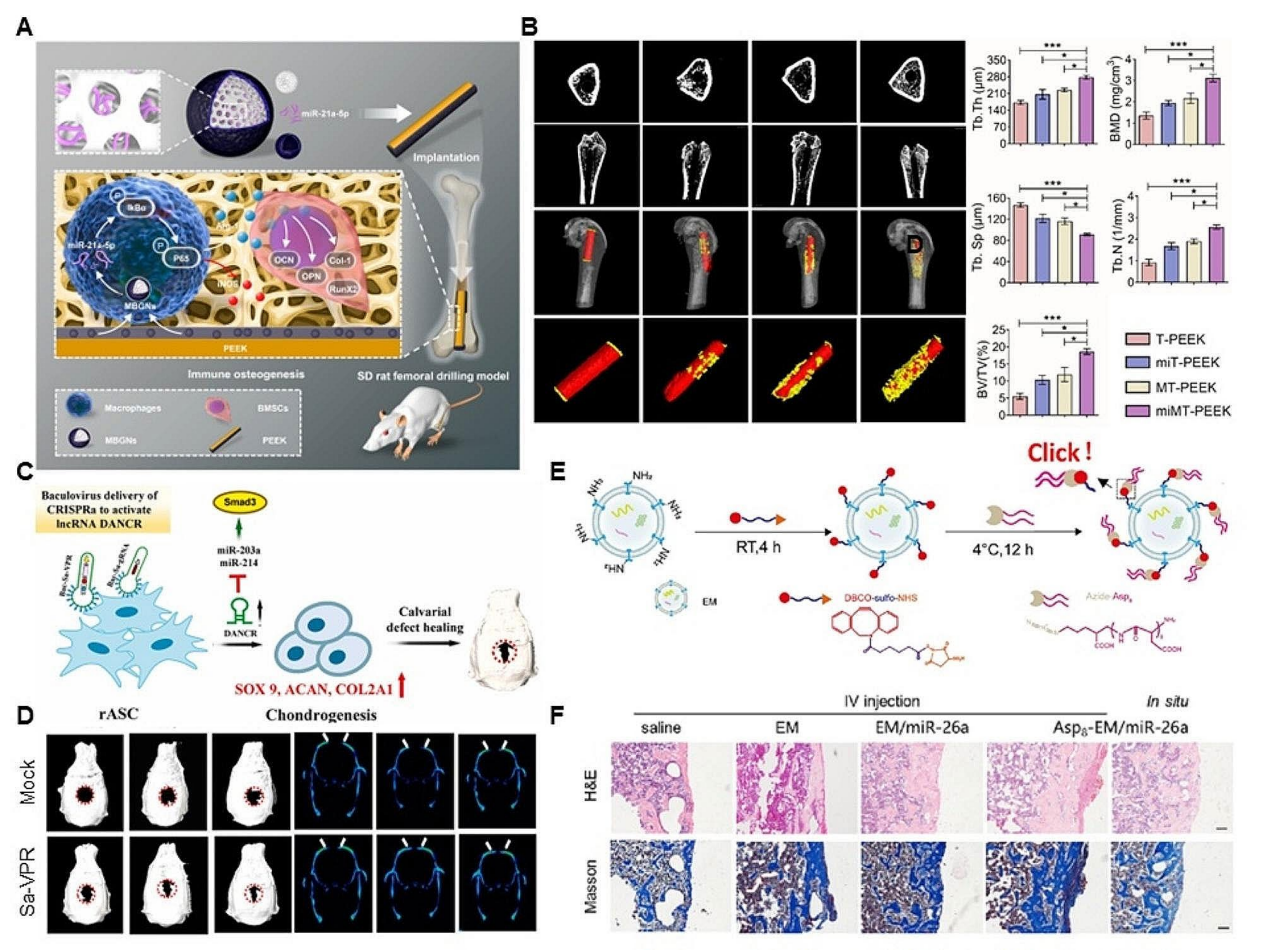
Biomaterials-based gene delivery vectors for bone regeneration. (**A**) Non-coding-RNA-activated core/chitosan shell nanounits coated with polyetheretherketone with the ability to promote bone regeneration. (**B**) Micro-CT images demonstrating bone regeneration surrounding implants, as well as reconstructed 3D images of samples and new bone. The color yellow denotes samples, whereas the color red represents fresh bone regeneration. Quantitative bone analysis 8 weeks after implantation. (**A**-**B**) Reproduced with permission [183]. Copyright 2023, American Chemical Society. (**C**) CRISPR activation of DANCR promotes calvarial bone formation. (**D**) µCT analysis of in vivo calvarial bone repair. 3D projection of osteomized sites of mock and Bac-Sa-VPR groups (left) and frontal views of osteomized sites (right). (**C**-**D**) Reproduced with permission [184]. Copyright 2021, Elsevier. (**E**) Schematic diagram of conjugating Asp8 to exosomal amine groups by click chemistry reaction. (**F**) H&E staining and Masson’s trichrome staining analysis of defect sites at 2 weeks, scale bar = 100 μm. (**E**-**F**) Reproduced with permission [185]. Copyright 2023, Elsevier

Advanced functional biomaterials can serve as gene delivery carriers for gene therapy in bone regeneration. Li and collaborators develop non-coding-RNA-induced core/chitosan shell nanounits to improve bone incorporation by immunoregulation (Fig. 5A). miMT-PEEK contributed macrophage M2 polarization via the NF-κB pathway to enhance BMSCs osteogenic differentiation. In vivo, miMT-PEEK increases osteogenesis and osseointegration, verified by Micro-CT images and histological images (Fig. 5B) [183]. Moreover, Hu and collaborators firstly verify that DANCR expression promotes rASC chondrogenesis. Then, the CRISPR activation (CRISPRa) technology is conducted to upregulate endogenous DANCR, promote rASC chondrogenesis and increase calvarial bone healing in rats (Fig. 5C). The µCT analysis in vivo also verifies the effect of DANCR NPs on calvarial bone repair (Fig. 5D) [184]. Further, Zhao and collaborators have designed Asp8-EM/miR-26a to enhance the synergetic effects for bone regeneration with increased targeting ability by Asp8 modification in bone defect and osteoporosis models (Fig. 5E-F) [185]. Therefore, the nonviral-mediated gene delivery system can regulate gene therapy ability in bone regeneration and make great achievement for clinical potential.

#### Advanced gene nanocarriers/scaffolds for gene therapy in cartilage regeneration

Advanced gene delivery biomaterials are applied to control targeting gene delivery efficiency into desired damaged sites for gene therapy and cartilage regeneration [186]. For instance, untreated damage to articular cartilage often results in osteoarthritis to induce inevitable disability [187]. Bone-marrow stimulation procedures, autologous or allograft, and periosteum transplantation are the main treatment methods in clinic for cartilage repair, for example, CuS/TGF-β1@PC NPs are prepared to deliver genes for cartilage regeneration [161, 188]. Nevertheless, these processes are usually accompanied by surgical trauma and poor long-term efficacy. Besides, the limited availability of donor sites and immune rejection may also restrict the broad application of gene therapy in cartilage regeneration [189]. Therefore, new comprehensive cartilage repair strategies are urgently needed based on efficient gene delivery and therapy. Advanced biomaterials-guided gene delivery provides the novel solution for precise delivery to direct targeted gene therapy [190]. Up to now, inorganic NPs, liposomes and cationic polymers are the commonly used biomaterials for gene delivery in cartilage regeneration [190]. These NPs can spatiotemporally enhance the process of cartilage repair by improving gene delivery ability [191]. Based on the superiority, the designed strategies of different biomaterials are also introduced and their important role is highlighted in gene therapy for cartilage repair.

**Fig. 6:**
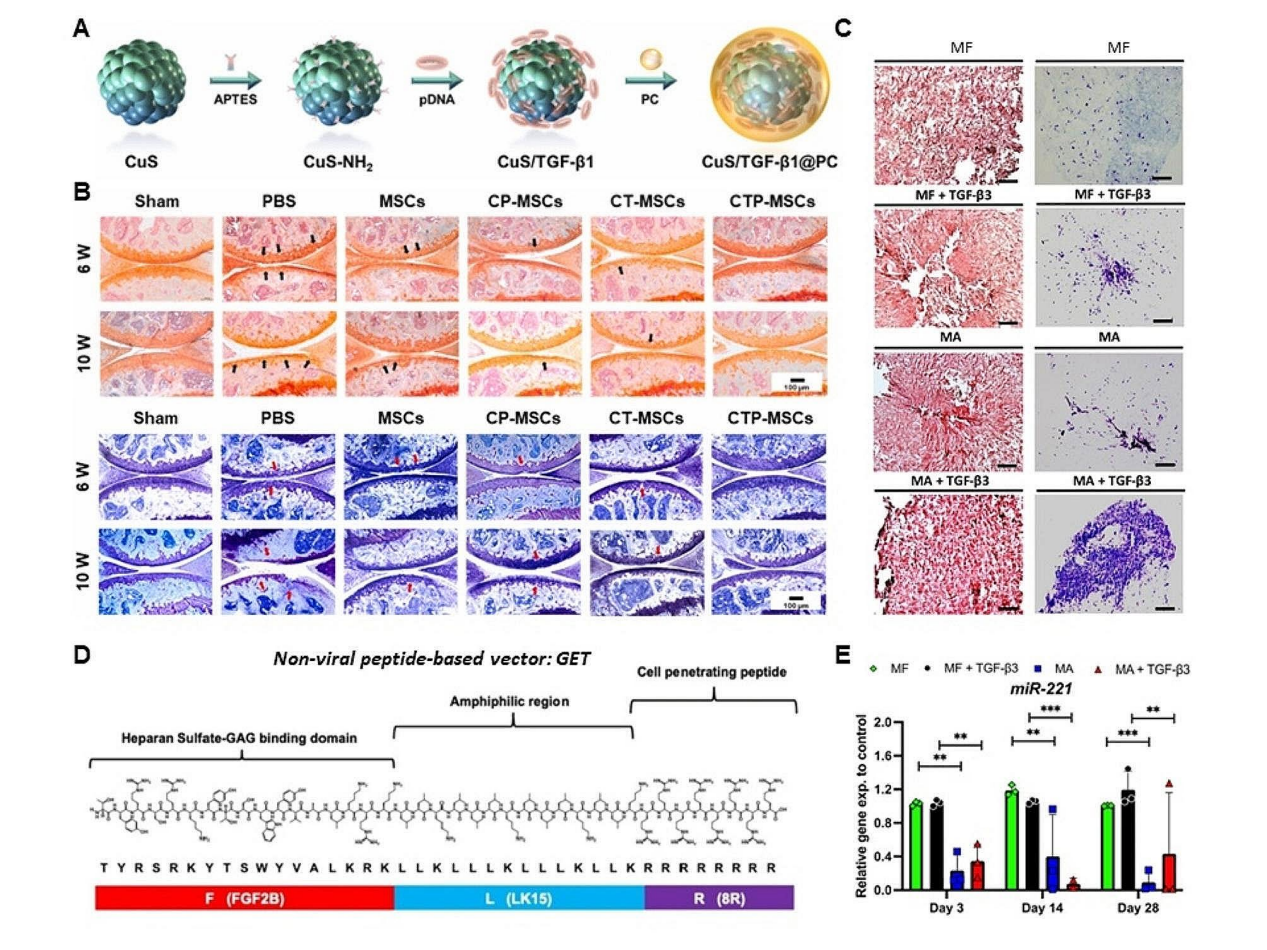
Biomaterials-based gene delivery vectors for cartilage regeneration. (**A**) Schematic illustration of the preparation of the Cu-based NPs. CuS NPs are reacted with ATPES to obtain CuS-NH_2_ NPs, and then electrostatically bonded with negatively charged TGF-β1 pDNA. Then PC is coated on the NPs for biomimetic modification. (**B**) Representative images of Safranin-O/Fast green staining and Toluidine blue staining of the knee joints after different treatments. (**A**-**B**) Reproduced with permission [162]. Copyright 2021, Elsevier. (**C**) The silencing of miR-221 in combination with TGF-β3 improved sGAG distribution. Representative histological images of cell-seeded scaffolds stained with (**A**) hematoxylin and eosin and (**B**) thionine after 28 days in culture +/− TGF-β3 supplementation. Scale bars represent 100 μm length. (**D**) Representation of GET peptide: a glycosaminoglycan (GAG)-binding peptide sequence (P21), fused to an amphiphilic region with an octa-arginine (8R) and a cell-penetrating peptide (CPP). (**E**) The incorporation of miR-221 inhibitor to CI/II-HyA scaffolds reduced the expression of miR-221 by hMSCs. (**C**-**E**) Reproduced with permission [200]. Copyright 2021, Wiley

Inorganic NPs occupy an important position in gene delivery and therapy for cartilage regeneration [192]. Cai and collaborators have designed and synthesized the biomimetic cupper sulfide@phosphatidylcholine (CuS@PC) NPs loaded with pDNA encoding transforming growth factor-beta 1 (TGF-β1) to operate MSCs for increased osteoarthritis treatment via cartilage regeneration (Fig. 6A, B) [162]. CuS/TGF-β1@PC NPs are more efficient than commercial transfection agent for gene delivery and present increased cell migration, chondrogenesis and inhibition of ECM degradation [162]. However, the low transfection efficiency and potential cytotoxicity restrict its further development and application in clinic. To detour these disturbances, liposomes have been widely recognized as a commercial transfection reagent for efficient gene delivery due to good biocompatibility [193]. At the same time, the influence of polymer-based carriers on gene delivery and therapy for cartilage repair are also studied and applied in gene delivery process [190]. For instance, hydrogels become research hot spot for gene delivery and therapy [194]. Dong and collaborators have developed an injectable chitosan/silk fibroin hydrogel for cartilage repair, which could release stromal cell-derived factor-1 (SDF-1) and kartogenin (KGN), in turn to enhance the recruitment and chondrogenic differentiation of MSCs spatiotemporally [195]. For autologous treatment, the poor regenerative ability of adult cells and the inflammatory state of the injured joint are the main obstacles. Bonato and collaborators have also encapsulated the TAK1-KO (TGF-β-activated kinase 1 knockout) chondrocytes into a hyaluronan hydrogel to deposit copious cartilage extracellular matrix proteins and facilitate integration onto native cartilage, even under proinflammatory conditions [163]. In another study, Chen and collaborators have used polyamidoamine dendrimer with amino acids as efficient vectors to deliver microRNA-224-5p (miR-224-5p), and the vector could condense miR-224-5p into transfected nanoparticles, which can show higher cell internalization and transfer ability compared to Lipofectamine 3000, and also protect miR-224-5p from RNase degradation to enhance miR-224-5p delivery ability [196].

Organic/inorganic nanohybrids also serve as multifunctional gene delivery systems in cartilage regeneration [197]. In particular, nanohybrids comprising of cationic polymers and inorganic NPs are considered promising candidates as advanced functional gene delivery system [198]. To promote gene delivery and therapy efficiency, glycosaminoglycan is designed as advanced gene delivery carriers for cartilage regeneration [199]. Claudio and collaborators have fabricated the microRNA (miR)-activated scaffolds, in which glycosaminoglycan-binding enhanced transduction (GET) NPs system is used to encapsulate the miR-221 inhibitor (Fig. 6C-E) [200]. The miR-activated scaffolds can successfully transfect human MSCs with the miR-221 cargos in a sustained and controlled manner up to 28 days, which is promising to enhance cartilage defect repair. Nucleic kinase substrate short peptide (pNNS)-conjugated chitosan (pNNS-CS) is also applied to improve gene delivery and therapy ability for cartilage repair [201]. Zhao and collaborators have reported the enhanced transfection efficiency of a pDNA-chitosan (pDNA-CS) complex using a phosphorylatable nuclear localization signal-linked nucleic kinase substrate short peptide (pNNS) conjugated to chitosan (pNNS-CS) [202]. On the other hand, they also investigate the effects of pNNS-CS-mediated miR-140 and IGF-1 in both rabbit chondrocytes and cartilage defects model. The results indicate that pNNS-CS serves as an excellent gene delivery vector in gene therapy for cartilage defects and that miR-140 combination with IGF-1 transfection has better biological effect on cartilage defects.

Exosomes are one of the most popular gene delivery vehicles, especially for cartilage regeneration and repair [203]. Mao and collaborators have suggested that exosomal microRNA-92a-3p (miR-92a-3p) can regulate cartilage development and homeostasis by directly targeting WNT5A [204]. Exosomal miR-92a-3p may act as a Wnt inhibitor and exhibit the potential as a disease-modifying osteoarthritis drug. In addition, Xu and collaborators have also reported that BMSC-Exos can deliver miR-326 to chondrocytes and cartilage to improve OA by targeting HDAC3 and STAT1/NF-kappa B p65 to inhibit pyroptosis of chondrocytes and cartilage [205].

Although biomaterial-based gene therapy has achieved great success in cartilage regeneration and repair, there are little nonviral vectors to achieve extremely high transfection efficiency [190, 206]. It is still an important task to improve transfection efficiency as much as possible on the premise of ensuring safety [190]. In addition, it is necessary to enhance the matching ability of gene release with the various stages of the chondrogenesis process [207]. Therefore, once these shortcomings are solved, it still can’t stop biomaterial-based gene therapy from representing the future direction for cartilage regeneration.

#### Advanced gene nanocarriers/scaffolds for gene therapy in blood vessel regeneration

Angiogenesis, the growth of new vessels, is a vital process for tissue regeneration of circulation research [208]. During the process of blood vessel regeneration, advanced biomaterial-based nonviral vectors play a vital role in regulating gene delivery and therapy. Among them, inorganic NPs, liposomes and cationic polymers are common gene carriers for blood vessel regeneration [52, 208].

**Fig. 7:**
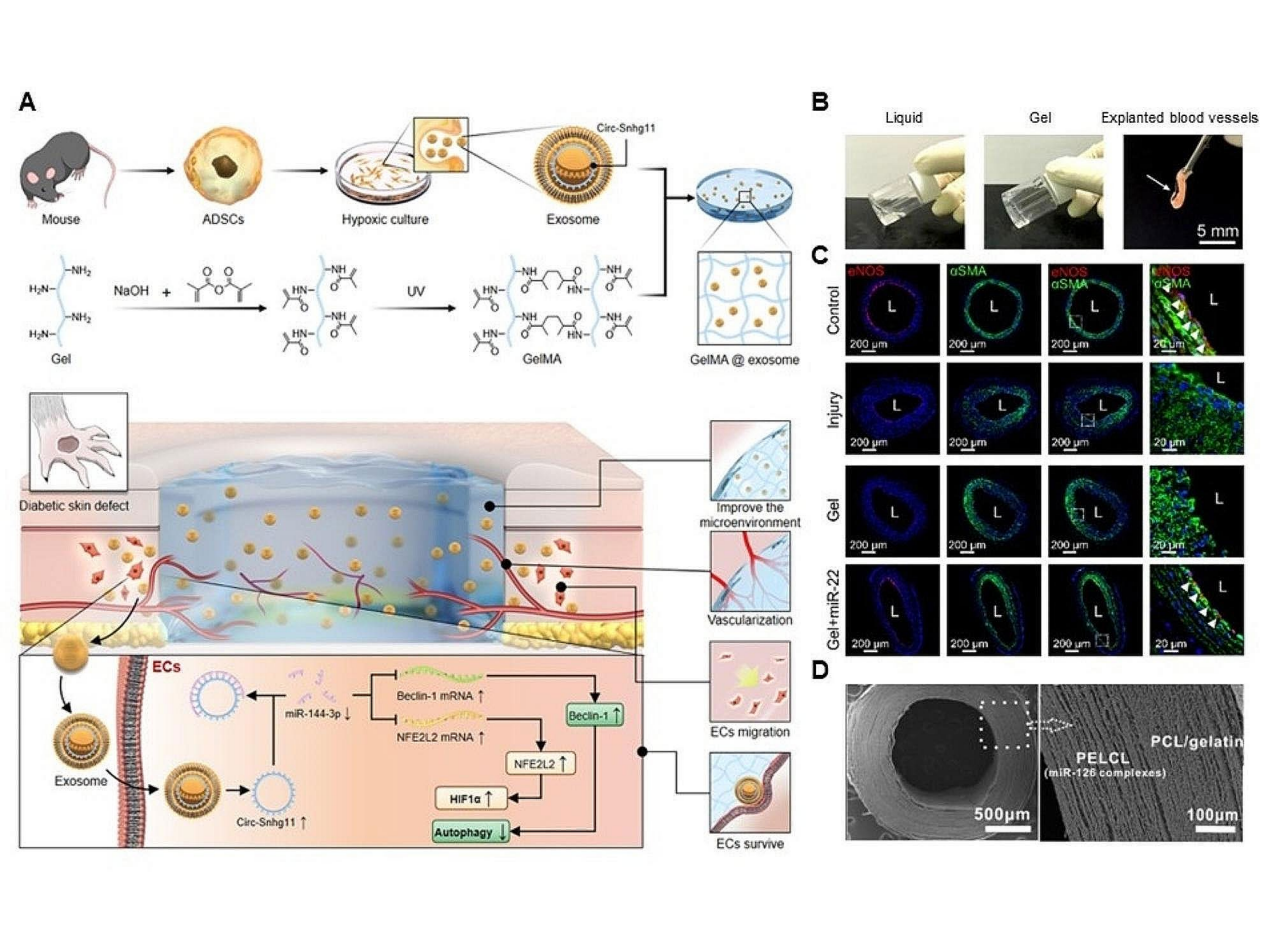
Biomaterial-based gene delivery vectors for blood vessel regeneration. (**A**) Hypoxia-pretreated ADSC-derived exosome-embedded hydrogels promote angiogenesis and accelerate diabetic wound healing. Reproduced with permission [166]. Copyright 2023, Elsevier. (**B**) The gelation of the laponite hydrogels in vitro. (**C**) Co-staining of the injured rat common carotid arteries with eNOS and αSMA 14 days after the treatment with the miR-22 loading laponite hydrogels. (**B**, **C**) Reproduced with permission [211]. Copyright 2022, Elsevier. (**D**) Morphology of the prepared vascular scaffold. SEM micrograph of the cross section and larger magnification. Reproduced with permission [214]. Copyright 2016, Elsevier

Mesoporous silica NPs are considered as gene therapy vectors and used in blood vessel regeneration [164]. To enhance delivery ability of mesoporous silica NPs, Wang and collaborators have investigated that mesoporous silica NPs-encapsulated miR-124 inhibitor increases the expression of SCF/c-kit protein by targeting P2Y(12) to improve the regeneration of cerebral blood vessels in lacunar cerebral infarction [165]. Although inorganic materials present the significant power in controlling gene delivery and therapy, the degradation and metabolic toxicity are still not negligible [209]. Alternatively, polymer-based gene therapy is showing huge advantages at this time. For instance, hydrogel scaffolds have the important influence on gene delivery [210]. Hu and collaborators have constructed exosome-embedded GelMA (GelMA-Exo) hydrogels with the ability of promoting diabetic wound healing, which could also deliver circ-Snhg11 and thereby increase the survival of endothelial cells to maintain endothelial cell (EC) function by activation of miR-144-3p/NFE2L2/HIF1α signaling pathway (Fig. 7A) [166]. Further, Zheng and collaborators have also found that the miR-22 loading laponite hydrogels inhibit the neointimal formation to reduce the infiltration of the macrophages and reverse the adverse vascular ECM remodeling by the upregulation of miR-22 and downregulation of its target genes methyl-CpG binding protein 2 (MECP2), which may offer a novel strategy to treat cardiovascular diseases (Fig. 7B, C) [211]. Polymer-based nanofibers also serve as a common delivery vector for gene delivery [212, 213]. Zhou and collaborators have fabricated a bilayer vascular scaffold *via* emulsion electrospinning of poly(ethylene glycol)-b-poly(l-lactide-co-ε-caprolactone) (PELCL), dual-power electrospinning of poly(ε-caprolactone) (PCL) and gelatin (Fig. 7D) [214]. The results demonstrate that the inner layer of PELCL loading with miR-126 could improve endothelialization ability in vivo. Local delivery of miRNA-based nanofibers may be an effective method to facilitate blood vessel regeneration [213, 215]. Maryam and collaborators have found that incubation of human endothelial cells (HUVECs) with polyhedral oligomeric silsesquioxane NPs (POSS NPs) could contribute to the modulation of angiogenesis and exosome biogenesis, which benefits to angiogenesis [216]. Some different types of polymer materials, for example, poly(L-lactic acid) (PLLA), poly(L-lactic acid)-co-poly(-caprolactone) [P(LLA-CL)] and eN-(2-hydroxypropyl)methacrylamide (HPMA) copolymer, also play a crucial role in regulating gene delivery and therapy for blood vessel regeneration [217, 218].

Polymer-based gene delivery materials can not only take part in gene delivery and therapy, but also control many other processes of endothelial cells, for example, nanocarriers with good biological activity due to surface and volume effects are more conducive to gene loading, cell adhesion and proliferation [219]. For hydrogel scaffolds, they can offer the possibility of sustained blood vessel regeneration [220]. Further, the binding and migration ability of transfected cells is limited in hydrogels for blood vessel regeneration [221]. Therefore, polymer-based materials allow the combination of multiple components and forms, and the integration of multiple materials has become the next development direction of biomaterial-mediated gene therapy.

#### Advanced gene nanocarriers/scaffolds for gene therapy in neuroregeneration

Peripheral nerve injuries (PNIs) are frequently encountered in clinical practice [222]. Short distant defected peripheral nerve can regenerate by repairing and suturing, but the repair of long distant nerve defect is still a global problem [223]. Currently, the autologous nerve graft is the gold standard treatment for long-distance nerve defect [223]. However, some challenges remain pendent for PNIs repair, such as sensory loss, scarring, and neuroma formation at the donor site [135, 167]. Moreover, size and fascicle mismatch, tubercle, and fibrosis also limit their applications in neuroregeneration [167]. To solve these problems, gene therapy has been applied in neuroregeneration and reported for nerve regeneration [224]. It is imminent to develop efficient biomaterial-based gene delivery vectors for high transfection efficiency of neuronal cells [225]. An overview of studies about neuronal cell transfection approaches, nonviral-vectors and their transfection efficiencies are summarized in Table 4.

**Table 4:** Nonviral-mediated delivery of nucleic acids for nerve system repair

Delivery methods	Therapeutic nucleic acids	Delivery vectors	Therapeutic outcomes	Ref.
Intrathecal administration	Plasmid DNA	Polyethylenimine (PEI)	Prolonged transgene expression in the spinal cord	[226]
Injection	MiR-221/222	Cationic polymer (PDAPEI)	Promoted nerve regeneration after sciatic nerve crush	[227]
Injection	siRNA	Chitosan/siRNA nanoparticle	A reduction of 65–75% of targeted mRNA and neurite outgrowth was improved	[228]
Scaffold implantation	MicroRNA-222	Chitosan nanoparticles with silk fibroin (SF) nanofibrous scaffolds	Enhance neuronal differentiation of neural stem cells	[229]
Intrathecal injection	mRNA	(PEG)-polyamino acid block copolymer	Sustained protein expression in the cerebrospinal fluid for almost a week	[230]
Tail vein injection	siRNA	Extracellular vesicles	Enhancing the therapeutic effects on SCI	[231]
In vitro transfection	miR-21a	Exosomes	Achieve their therapeutic effects in promoting neurogenesis	[168]
Subcutaneous injection	miR-100	Nanovesicles	Promotion hair follicle growth	[169]
Scaffold implantation	Small non-coding RNAs	Nanofibers-hydrogel scaffold	Aligned axon regeneration	[232]
Scaffold implantation	pDNA	biodegradable polycations	Enhancement recovery of hindlimb motor function	[233]
Intraocular injections	siRNA	polybutylcyanoacrylate nanoparticles (CaspNPs)	Restoration of central visual system damage	[234]
Intrathecal injection	Vector DNA	PEI/DNA polyplexes	Protein expression was detected in 82.8% ± 1.70% of DRG neurons	[235]

Liposomes as a commercial reagent work through the effects of cationic lipid molecules [236]. The molecules containing positive-charge head groups can interact with negative-charge nucleic acids to form the complexes [237, 238]. Then, the complexes fuse with the cell membrane and efficiently deliver the nucleic acids into cells for gene therapy [85]. Liposomes have low toxicity and stable transfection ability for gene delivery, and may be further applied for neuron regeneration and repair in vivo [89, 239].

Cationic polymers have caused intense attention in nerve regeneration [240]. Shi and collaborators have studied the effect of intrathecal administration of pDNA/PEI complex into the lumbar subarachnoid space and the result is satisfactory for high efficient gene delivery [226]. Some researchers have proposed the modification of PEI by polyethylene glycol (i.e. PEGylation) could improve biocompatibility [241]. Song and collaborators have reported a biodegradable and biocompatible cationic polymer (PDA/PEI) by cross-linking low molecular weight PEI (1.8 kDa) with 2,6-pyridinedicarboxaldehyde [242]. During the transfection process, PDA/PEI shows a lower cytotoxicity and higher transfection efficiency than PEI (25 kDa) in transfecting miR-221/222 into rat Schwann cells (SCs) [227]. Due to the toxicity of PEI and its modified polymers, natural polysaccharides (i.e. chitosan) are chosen as gene vectors for gene delivery and therapy [243]. Chitosan with biocompatibility, biodegradability and low toxicity have attended more and more attraction in gene delivery for nerve regeneration [244, 245]. Mittnacht and collaborators have fabricated the polymer filaments loaded with chitosan/siRNA nanoparticles to promote nerve regeneration. There is a reduction of 65–75% of targeted mRNA and neurite outgrowth is improved [228]. Scaffold-mediated nonviral gene delivery for nerve regeneration also takes an important position in regulating gene therapy [243]. Li and collaborators have incorporated the microRNA-222 loading chitosan NPs (miR-222/CS NPs) with silk fibroin (SF) nanofibrous scaffolds to enhance neuronal differentiation of neural stem cells [229]. Sustained release and delivery of miR-222 in SF-based scaffold can improve the chance of successful gene transfection.

## Gene therapy for cancer-resected tissue repair

### Cancer gene therapy

**Fig. 8:**
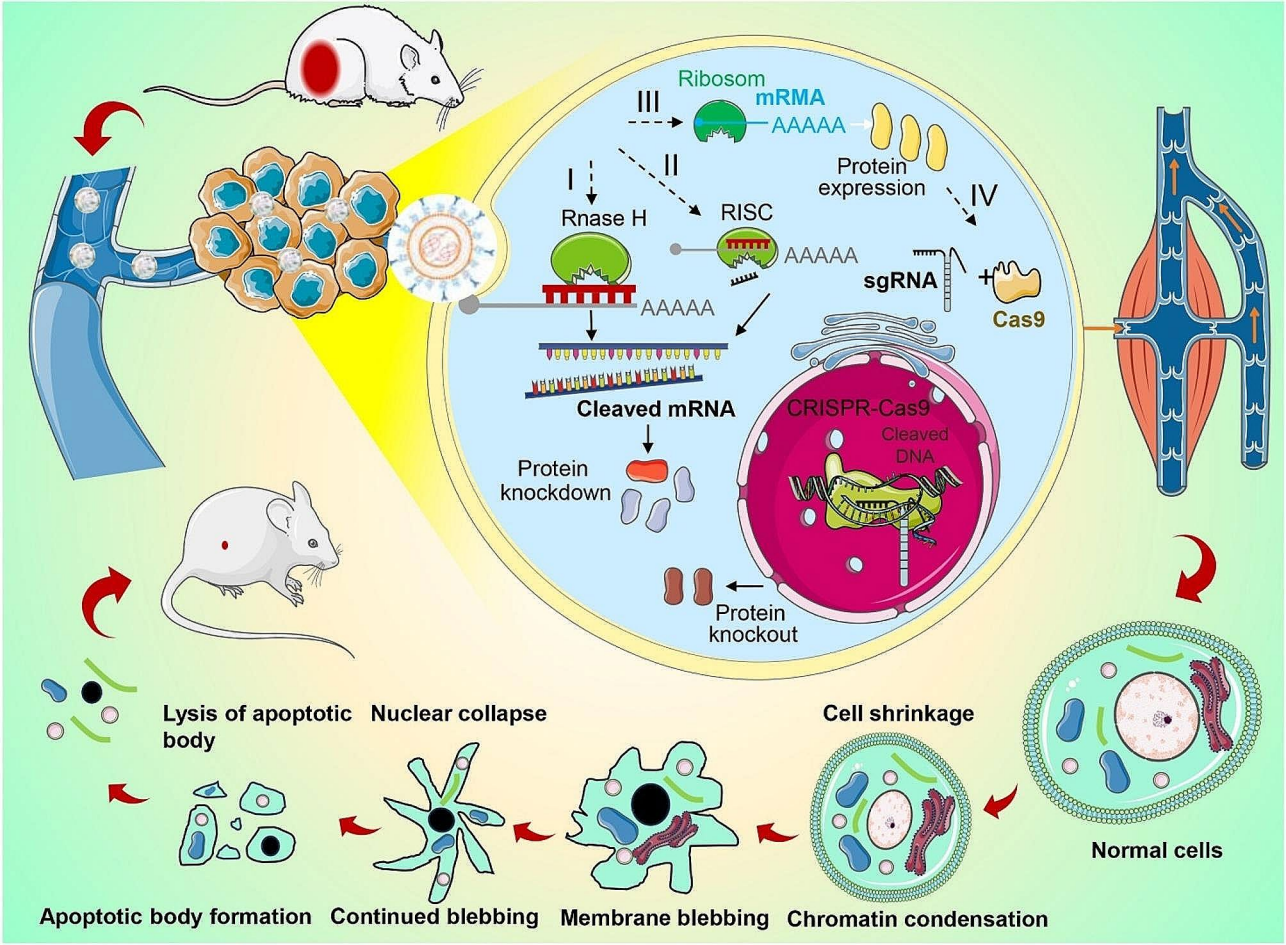
Regulation of gene and protein expression using RNA/Nucleic acid in cancer therapy for tissue regeneration. Once delivered into the cells, RNA macromolecules can utilize diverse intracellular mechanism to control gene and protein expression in cancer cells for gene therapy. (I) Hybridization of antisense oligonucleotides (ASOs) to a targeting mRNA can result in specific inhibition of gene expression by the induction of RNase H endonuclease activity, which cleaves the mRNA-ASO heteroduplex. (II) Short interfering RNA (siRNA) is recognized by the RNA-induced silencing complex (RISC), guided by an antisense strand of the siRNA, specifically to bind and cleave targeting mRNA. (III) In vitro transcribed mRNA utilizes the protein synthesis machinery of host cells to translate the encoded genetic information into a protein. Ribosome subunits are recruited to mRNA with a cap and poly(A)-binding proteins, forming a translation initiation complex. (IV) In the clustered regularly interspaced short palindromic repeats (CRISPR-Cas9) system, co-delivery of a single guide RNA (sgRNA) together with the mRNA encoding the Cas9 DNA endonuclease allows site-specific cleavage of dsDNA, leading to the knockout of a target gene and its product. Then, this will realize cancer therapy by RNA/Nucleic acid delivery

Cancer is an unsolved worldwide problem due to high morbidity and mortality [246]. Cancer gene therapy refers to the delivery of therapeutic genes to target cells through appropriate carriers to precisely control expression up-regulation/down-regulation of specific tumor-related genes, so as to achieve efficient cancer treatment [247, 248]. The regulation process of gene and protein expression using RNA/nucleic acid in cancer therapy is essential for cancer-resected tissue repair (Fig. 8). Once genes are delivered into the targeting cells, RNA macromolecules can utilize diverse intracellular molecules to adjust gene and protein expression [249]. Then, the tumor suppressor gene is highly expressed and acts on cells to induce tumor cell apoptosis for cancer gene therapy. After the treatment of cancers, a tissue defect is exposed and it is difficult to heal damaged tissue [250, 251]. Therefore, gene therapy for cancer-resected tissue repair is demanded to coordinate tumor treatment and tissue regeneration [252, 253].

To enhance the cancer therapy efficiency and tissue repair ability, many researchers pay more attention on the inorganic NPs as gene delivery vectors for gene therapy [254]. Cristofolini and collaborators have fabricated a multifunctional hybrid NPs as magnetic delivery system for siRNA targeting the HER2 gene in breast cancer cells for efficient gene therapy and wound healing [255]. Kara and collaborators have also developed a novel poly-L-lysine (PLL)-modified sericin-coated superparamagnetic iron oxide NPs (PLL/Ser-SPIONs) as siRNA carriers [256]. PLL/Ser-SPIONs can act as a safe and promising carrier candidate to deliver siRNA-based genes into cells to treat cancer cells and induce tissue development for further applications in gene therapy and tissue engineering [256]. However, the major disadvantages of inorganic NPs are non-degradable nature, and entrapment by the lungs, liver, and kidneys, which is difficult for in vivo gene therapy in clinic [257].

In addition of inorganic NPs, the commonly used positive-charge polymers including PLL, PEI, polyamide (PAMAM), and poly(β-amino ester) (PBAE), which can interact with the negative-charge phosphate groups on the main chain of RNA and form complexes into cells and realize escape of RNA-bound endosomes [258–261]. Among them, PEI serves as a classic and effective transfection agent for gene therapy in cancer and tissue regeneration [99, 101–103]. In addition, PEI has a high buffering capacity in a very wide pH range. Chen and collaborators have reported the cationic perfluorocarbon nanoemulsions with positive surface charge provided by a fluorinated PEI (F-PEI) [262]. The fluorinated emulsion (F-PEI@PFD) with reduced cytotoxicity can enhance cellular uptake and improve endosomal escape of the siRNA for gene delivery and therapy in cancer-resected tissue engineering.

EVs are small membrane vesicles released into the extracellular matrix and they also play an important role in gene delivery and therapy in cancer [59]. Mutifunctional exosomes are derived from different cells types, including cancer cells, dendritic cells, B cells, T cells, mast cells and epithelial cells to deliver targeting genes for cancer therapy and tissue repair [63–66]. For example, HEK293 cells with advantages of high transfection and easy to gene operation are allowed for gene manipulation on exosomes. The biggest advantage of cancer cell-derived exosomes involved in gene delivery is targeting and low immunogenicity [263]. Zhou and collaborators have reported a novel mechanism that targeting tumor exosomal circular RNA cSERPINE2 suppresses breast cancer progression by mediating MALT1-NF-𝜅B-IL-6 axis of tumor-associated macrophages [264]. Ellipilli and collaborators have also reported GalNAc decorated exosomes as cargo for targeted delivery of Paclitaxel (PTX) and miR122 to liver tumors as an effective means to inhibit the hepatocellular carcinoma [265]. Because of the excellent performance, it has also been applied in cancer clinical trials. Researchers can consult related data on the database www.ClinicalTrials.gov (accessed in April, 2022) [266, 267]. However, some limitations are still remained for cancer-induced tissue repair, such as pharmacokinetic characteristics and potential safety issues [268–270].

Protein or peptide with unique sequences can demonstrate a variety of properties, including siRNA binding, membrane penetration, endosomal destruction, targeting, etc [271]. Cell-penetrating peptides (CPPS) are amphiphilic oligomers with positive-charge residues (arginine or lysine) and it is usually applied in gene delivery [272]. Deng and collaborators have reported a transdermal peptide, #PKU12. The antitumor effect of PKU12-based siRNA against HPV is perfect for human papillomavirus in vivo [273]. Protein or polypeptide delivery RNA strategies have high selectivity and activity in gene therapy and tissue repair.

### Cancer-resected gene therapy for tissue repair

**Fig. 9:**
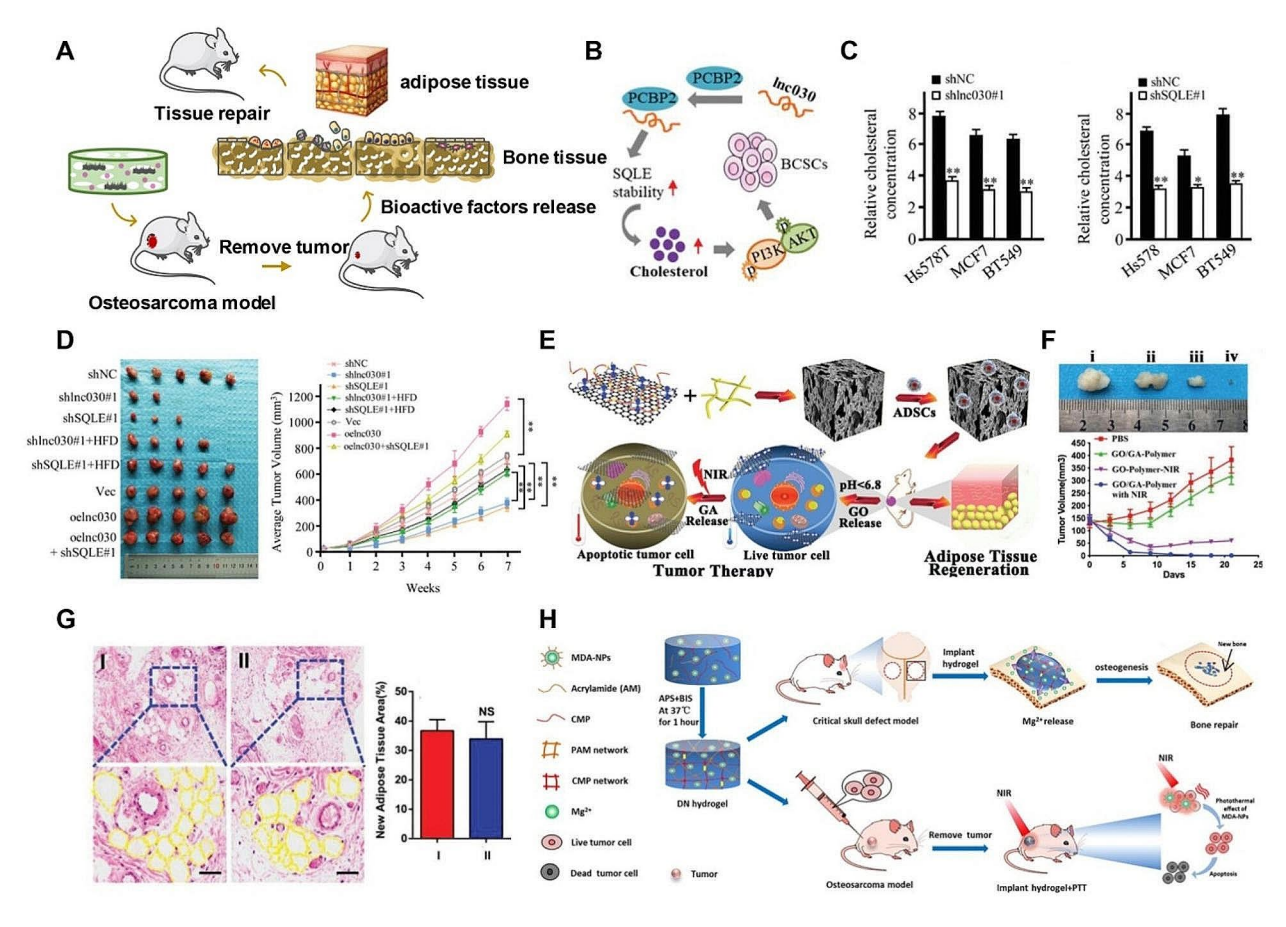
Regulation of gene and protein expression using RNA/Nucleic acid in cancer therapy for tissue regeneration. (**A**) A schematic diagram of gene therapy in tissue repair after removing tumors. (**B**) A schematic diagram to depict the role of lnc030 in maintenance of BCSC stemness through regulating SQLE and cholesterol accumulation to stimulate PI3K/Akt signaling. (**C**) Intracellular cholesterol is measured in lnc030-knockdown (left) or SQLE-knockdown (right) Hs578T, BT549, MCF-7 derived spheres and their control spheres. (**D**) Tumor growth curve and tumor weight of each mouse group are measured. (**B**-**D**) Reproduced with permission [275]. Copyright 2020, Wiley. (**E**) Schematic illustration of therapeutic process of ADSC-loaded GO-GA-polymer scaffold with pH-triggered dual release of GO and GA. (**F**) The tumor growth curves after implantation of different formulations with and without NIR irradiation. (**G**) Histological analysis and histomorphometric measurements of adipose tissue regeneration in vivo. (**E**-**G**) Reproduced with permission [276]. Copyright 2019, Wiley. (**H**) A schematic diagram of nanocomposite multifunctional hydrogel for suppressing osteosarcoma recurrence and enhancing bone regeneration. Reproduced with permission [274]. Copyright 2022, Elsevier

As mentioned before, tissue defects after tumor surgery are difficult to repair [277]. For breast cancer patients, surgical removal of tumor tissue usually results in permanent breast defects, which brings great psychological pain to the patients [278]. Therefore, gene therapy plays an important role to induce post-cancer repair and tissue regeneration (Fig. 9A) [279]. Liu and collaborators report that a long non-coding RNA lnc030, cooperated with poly(rC) binding protein 2(PCBP2) to stabilize squalene epoxidase (SQLE) mRNA, resulting in an increase of cholesterol synthesis (Fig. 9B) [275]. The increased cholesterol also activates PI3K/Akt signaling and governs breast cancer stem cells stemness. Therefore, the lnc030-SQLE-cholesterol synthesis pathway is a therapeutic target for breast cancer treatment and adipose tissue regeneration (Fig. 9C, D) [275].

In addition, advanced functional scaffolds are also applied for cancer-resected tissue regeneration. Chen and collaborators fabricate a stimuli-responsive scaffold for breast cancer treatment and adipose tissue regeneration (Fig. 9E). This scaffold is composed of polyacrylic acid-g-polylactic acid (PAA-g-PLLA) modified graphene oxide (GO) with a cleavable bond in between (GO-PAA-g-PLLA), gambogic acid (GA), and polycaprolactone (PCL). These complex scaffolds can not only selectively induce the apoptosis of tumor cells, but also improve the capability to stimulate the differentiation of ADSCs into adipocytes (Fig. 9F, G) [276]. Chen and collaborators have also fabricated a composite scaffold of gelatin and Fe_3_O_4_ NPs for magnetic hyperthermia-based breast cancer treatment and adipose tissue regeneration. It can ablate breast cancer cell and facilitate the growth and adipogenesis of mesenchymal stem cells [280]. Currently, photothermal therapy is the commonly used method for most scaffolds for breast tumor treatment and adipose tissue regeneration [281]. If gene therapy is integrated into these scaffolds, better results will be achieved for adipose regeneration. The scaffolds with RNA lnc030 integration can realize breast cancer treatment and further contribute to the adipose tissue regeneration.

Similarly, bone tumor is clinically a malignant tumor and surgical resection is the common clinical treatment [282]. Although native bone tissue has the self-regeneration ability, inevitable damage to the bone is still unsettled [283, 284]. Therefore, gene therapy would be a preferable strategy to introduce targeting genes into cells for bone tumor treatment and bone tissue regeneration. Zhang and collaborators have fabricated a functional nanovector to deliver astrocyte elevated gene-1 (AEG-1) small interfering RNA (siRNA; siAEG-1) into osteosarcoma cells to silence the targeted gene both in vitro and in vivo. The results show that the Cs-g-PLLD-FA/siAEG-1 nanocomplexes could knockdown AEG-1 genes to inhibit the tumor cell proliferation, invasion and lung metastasis in tumor-bearing mice with low cytotoxicity and high efficacy [285]. Nasr-Esfahani and collaborators also prepare mesoporous bioactive glass (MBG) sub-micro particles to present the potential in treatment of osteosarcoma by releasing bioactive ions of Ca, Si and P, and contribute to bone regeneration by depositing hydroxylapatite (HA) [286]. Lai and collaborators demonstrate that multifunctional nanocomposite hydrogel has an excellent effect for suppressing osteosarcoma recurrence and enhancing bone regeneration (Fig. 9H) [274].

At present, many researchers have proposed the gene therapy method to treat bone tumor by preparing high-efficacy and low-toxicity vectors to discover novel long non-coding RNA, circular RNA or miRNA [287, 288]. Hu and collaborators have even mapped the single-cell RNA landscape of intratumoral heterogeneity and immunosuppressive microenvironment in advanced osteosarcoma, which provides potential therapeutic targets for osteosarcoma [288]. Moreover, there are also many novel methods to treat bone tumor and contribute to bone regeneration by fabricating multifunctional scaffolds or vectors [274, 289, 290]. Combining gene therapy vectors and bioactive scaffolds, advanced biomaterials will provide a promising insight for synergetic tumor treatment and tissue regeneration (Table 5).

**Table 5:** Cancer-resected gene therapy for tissue repair

Therapeutic nucleic acids	Delivery vectors	Therapeutic outcomes	Reference
lnc030	poly(rC) binding protein 2(PCBP2)	A therapeutic target for breast cancer treatment and adipose tissue regeneration	[269]
siAEG-1	Nanocomplexes	Knockdown AEG-1 genes to inhibit the tumor cell proliferation, invasion and lung metastasis in tumor-bearing mice with low cytotoxicity and high effiency	[280]
branched-DNA	DNA-polyphenol nanocomplex	Showed specific targeting to tumor and achieved the best medical intervention effects	[291]
branched-DNA/RNA	nanocomplex@A549m)	effectively delivered therapeutic siRNA to tumor sites and exhibited high RNAi efficiency and enhanced anti-tumor activity	[292]
siRNA	siRNA/EGCG/protamine/HA	displayed the highest tumor inhibition effect and little toxicity to normal tissues and organs	[293]
siRNA	MNC@LPMSA@siRNA@TA	siRNA was effectively delivered into the cytoplasmic region	[294]
siEIF5A2	Mg(II)-Cat NPs	effectively accumulated in tumors and exerted significant antitumor effects via inhibiting phosphoinositide 3-kinase/protein kinase B (P13K/Akt) signaling pathway	[295]
siRNA	siRNA-functionalized DBC-FeIII MPN	In PC3-Luc2 cells, 89% of the luciferase gene was silenced by siRNA-functionalized DBC-FeIII MPN nanoparticles	[296]
siRNA	siRNA-functionalized DBC-FeIII MPN	89% of the luciferase gene was silenced by siRNA-functionalized DBC-FeIII MPN nanoparticles	[297]
miRNA-21	Hydrogel&MIC@Antagomir-21	sustained release of Antagomir-21 and on-demand release of Cur	[298]
dsRNA	NPPLL/EGCG/dsRNA	effectively protect dsRNA from double-stranded ribonuclease (dsRNase) degradation, increase the tolerance of dsRNA to dsRNase, enhance cellular uptake and endosome escape	[299]

## Conclusions and future perspectives

Tissue repair and regeneration are long-term concerns for postoperative patients, especially after orthopedics, neurology, and cancer operations. In order to alleviate the burden on patients and expedite recovery, regional gene therapy has been developed and is being considered as a potential approach for tissue regeneration and repair. Over the past two decades, regional gene therapy has been rapidly developed for tissue regeneration, and encompassing advances in vector types, delivery methods, as well as the design and fabrication of novel materials are also prosperous for efficient gene delivery and therapy in bone regeneration, cartilage regeneration, blood vessel regeneration, nerve regeneration and even cancer-resected tissue repair. Although gene therapy has shown the explosive development for tissue regeneration and repair, current mainstream programmes for regional gene therapy rely on viral-based vectors. Despite their inherent advantages such as extended duration and high payload capacity, there are still concerns about their safety, particularly in terms of immunogenicity. To completely overcome potential risks from viral and further improve gene delivery ability, various gene delivery biomaterials have been exploited for tissue regeneration and repair, including lipids, cationic polymers, peptides or proteins, dendritic/branched biomaterials, inorganic NPs and their composites. These novel gene carriers can not only enhance targeting accuracy and delivery ability to replace defective genes in damaged sites, but also emphasize the creation of new drugs and treatment procedures that are effective and less painful to eradicate all kinds of genetic tissue diseases. To date, gene therapy is still considered as the most potential solution to tissue regeneration and repair.

Improving the transfection efficiency, enhancing the transfection stability and reducing the toxicity are the common development direction of these biomaterials-based nonviral vectors for gene therapy and tissue regeneration. It is essential to further enhance the comprehension of the molecular mechanisms underlying tissue reconstruction and the effects of polymeric biomaterials on cell fate. Maximizing the transfection capacity of polymeric biomaterials in gene therapy remains a challenge, as currently, nonviral vectors are unable to achieve the same high transfection efficiency as viral vectors. In addition, although polymeric biomaterials offer desired release kinetics and spatially defined architectures, the optimal release time and the dose of therapeutic genes required for effective cartilage repair are still unknown. With the development of advanced biofabrication techniques, the combination of various biomaterials to construct composites with tailorable properties and spatially controlled biological function will be realized, and more personalized polymeric biomaterial-mediated gene delivery systems should be explored to promote clinical treatment effect. The precise regulation of each stage matching to the process of tissue formation is still hardly achieved at present. Therefore, advanced biomaterial-mediated gene delivery with efficient, well-defined and targeting transduction system will represent the next frontier to enhance tissue regeneration and repair in vivo.

## Data Availability

The data that support the findings of this study are available from the corresponding authors upon reasonable request.
