# Correction: Advanced gene nanocarriers/scaffolds in nonviral-mediated delivery system for tissue regeneration and repair

**DOI:** 10.1186/s12951-024-02769-x

**Published:** 2024-08-29

**Authors:** Wanheng Zhang, Yan Hou, Shiyi Yin, Qi Miao, Kyubae Lee, Xiaojian Zhou, Yongtao Wang

**Affiliations:** 1 Institute of Geriatrics, School of Medicine, Affiliated Nantong Hospital of Shanghai University (The Sixth People’s Hospital of Nantong), Shanghai University, Shanghai, 200444 China; 2 Joint International Research Laboratory of Biomaterials and Biotechnology in Organ Repair (Ministry of Education), Shanghai University, Shanghai, 200444 China; 3 Department of Pharmacy, China Pharmaceutical University, Nanjing, 210009 China; 4 Department of Biomedical Materials, Konyang University, Daejeon, 35365 Republic of Korea; 5 Department of Pediatrics, Shanghai General Hospital, School of Medicine, Shanghai Jiao Tong University, Shanghai, 200080 China

**Correction: Journal of Nanobiotechnology (2024) 22:376**

10.1186/s12951-024-02580-8

The authors of this article would like to update the figure caption for Figs. 6 and 9.

In Fig. 6, “(A-B) Reproduced with permission [161]. Copyright 2021, Elsevier.” should be changed to “(A-B) Reproduced with permission [162]. Copyright 2023, Elsevier”.

In Fig. 6, “(C-E) Reproduced with permission [163]. Copyright 2021, Wiley” should be changed to “(C-E) Reproduced with permission [200]. Copyright 2023, Wiley”.

Additionally, in “Advanced gene nanocarriers/scaffolds for gene therapy in cartilage regeneration” part of main text, “(Fig. 6C-D)” should be removed where reference 163 is cited, and “(Fig. 6C-E)” should be added where reference 200 is cited.

In Fig. 9A, the statement of “Reproduced with permission [274]. Copyright 2015, Elsevier.” should be removed as this has been organised by the authors. The original article has been corrected.

